# Somatic mutations render human exome and pathogen DNA more similar

**DOI:** 10.1371/journal.pone.0197949

**Published:** 2019-05-01

**Authors:** Ehsan Ebrahimzadeh, Maggie Engler, David Tse, Razvan Cristescu, Aslan Tchamkerten

**Affiliations:** 1 Department of Electrical Engineering, UCLA, Los Angeles, California, United States of America; 2 Department of Electrical Engineering, Stanford University, Stanford, California, United States of America; 3 Department of Discovery Medicine, Merck Research Laboratories, Rahway, New Jersey, United States of America; 4 Department of Communications and Electronics, Telecom ParisTech, Paris, France; CNR, ITALY

## Abstract

Immunotherapy has recently shown important clinical successes in a substantial number of oncology indications. Additionally, the tumor somatic mutation load has been shown to associate with response to these therapeutic agents, and specific mutational signatures are hypothesized to improve this association, including signatures related to pathogen insults. We sought to study in silico the validity of these observations and how they relate to each other. We first addressed the question whether somatic mutations typically involved in cancer may increase, in a statistically meaningful manner, the similarity between common pathogens and the human exome. Our study shows that common mutagenic processes like those resulting from exposure to ultraviolet light (in melanoma) or smoking (in lung cancer) increase, in the upper range of biologically plausible frequencies, the similarity between cancer exomes and pathogen DNA at a scale of 12 to 16 nucleotide sequences (corresponding to peptides of 4 – 5 amino acids). Second, we investigated whether this increased similarity is due to the specific mutation distribution of the considered mutagenic processes or whether uniformly random mutations at equal rate would trigger the same effect. Our results show that, depending on the combination of pathogen and mutagenic process, these effects need not be distinguishable. Third, we studied the impact of mutation rate and showed that increasing mutation rate generally results in an increased similarity between the cancer exome and pathogen DNA, again at a scale of 4 – 5 amino acids. Finally, we investigated whether the considered mutational processes result in amino-acid changes with functional relevance that are more likely to be immunogenic. We showed that functional tolerance to mutagenic processes across species generally suggests more resilience to mutagenic processes that are due to exposure to elements of nature than to mutagenic processes that are due to exposure to cancer-causing artificial substances. These results support the idea that recognition of pathogen sequences as well as differential functional tolerance to mutagenic processes may play an important role in the immune recognition process involved in tumor infiltration by lymphocytes.

## Introduction

Recent clinical advances firmly establish the role of immunotherapy (in particular, checkpoint inhibition targetting the CTLA4 and PD1/PD-L1 pathways [1]) in the treatment of cancer. However, the rates of response vary by indication, outlining the important role of identifying the patients most likely to respond [2–5]. In parallel, the analysis of the data in large scale genomic efforts including The Cancer Genome Atlas (TCGA [6]) has identified universal characteristics of the tumor and its environment that ellicit potential recognition by the host immune system. In particular, somatic mutational load as inferred by DNA sequencing [7, 8] and cytolytic infiltrate as inferred by immunohistochemistry or RNA sequencing [9] have emerged as hallmarks of an immune-active tumor enviroment. It is thus important to understand the causality and mechanism of action that drives the heterogenous composition of the tumor and its environment and consequently the heterogeneity of response to immunotherapy, in order to select the right patients for treatment, potential combinations, and potential for early intervention.

Multiple recent studies have suggested a strong causal link between the mutational burden of the tumor and clinical response to immunotherapy across multiple indications including Melanoma [10, 11], Non Small Cell Lung Cancer [12], Bladder cancer [13] and Colorectal cancer [14]. In these studies, a strong relationship between neoantigen load (the number of mutations with immunogenic potential) and response to immunotherapy has been identified. Importantly, each of these indications are characterized by distinct mutagenic processes that result in abundant neoantigen load [7, 8]: UV light exposure in Melanoma, smoking in Non Small Cell Lung Cancer, APOBEC activation in Bladder cancer, and MMR defficiency in MSI-h Colorectal cancer. Whether particular mutations or mutational patterns preferentially induce an immunologic phenotype remains an open question [10, 11]. However, several hypotheses have recently been put forward, including the presence of mutations in particular genes [15, 16], or the presence of a transversion signature related to smoking [12]. In particular, Snyder *et al*. [11] put forward a hypothesis linking cancer exomes with patterns present in common pathogens. Namely, their results with exome analysis of Melanoma patients treated with Ipilimumab, a CTLA4 inhibitor, suggest that somatic mutations in cancer genomes that lead to tetrapeptides similar to those found in common pathogens are more likely to elicit a response to the therapy than common somatic mutations. This association is presumably driven by the innate ability of significant portions of the adaptive immune repertoire to recognize such pathogens.

We took an in-silico approach to evaluate the impact of certain mutagenic processes on the similarity between cancer exomes and pathogen DNAs. Somatic mutations are an inherent natural process related to cell division and aging which in some instances is exacerbated by mutagenic factors. We simulated such mutagenic processes using mixtures of mutational signatures with empirically derived mixing parameters. We used a simple similarity metric between the mutated exome and common pathogen exomes to estimate changes in overall potential immunogenicity of cancer exomes as compared to the normal exome. We considered simulations of mutagenic processes that yield most mutated cancer exomes, namely ultra-violet (UV) light (Melanoma), smoking (Non Small Cell Lung Cancer), and APOBEC activation (Bladder cancer) [7, 9]. Our results suggest that, in the upper range of biologically plausible mutation rates, mutagenic processes resulting from exposure to these common mutagens lead to cancer exomes that are more similar pathogen DNAs at a scale of 12 to 16 nucleotides. These changes are subtle but nevertheless statistically significant and are particularly important in the range of peptide sizes that are relevant for epitope presentation in the human MHC mechanism; MHC presentation typically involves peptides with lengths between 8-18 nucleotides (8-13 for class I MHC and 13-18 for class II MHC [17]).

However, our results also suggest that the increased similarity need not be caused by the specificity of the mutation distribution. Depending on the pathogen, uniformly random mutations (at the same rate) may result in equal increased similarity. Finally, we show that increasing mutation rate generally results in increased similarity between cancer exomes and pathogen DNAs. These conclusions suggest that mutagenic processes might act as a mechanism of pressure that models the mutational spectra observed in tumors by increasing recognition from the host immune system.

Opposite to the aforementioned effect that increases the likelihood that a cancer exome is recognized by the immune system, an antagonist mechanism of pressure on mutational landscape stems from tolerance by the immune system to natural mutagenic processes. To that extent, we establish that exomes across species are generally more resilient, in terms of a functional point of view related to the synonymity of amino-acid changes, to mutagenic processes that are due to exposure to elements of nature than to mutagenic processes that are due to exposure to cancer-causing artificial substances. In particular, we observe that the functionality of the genetic code (allocation of codons to amino-acids) is more resilient to UV light than smoking mutagenic processes at a fixed rate. This suggests the possibility that there are different tissue-dependent evolutionary tolerance levels, modulated by the pathogen recognition apparatus in terms of both immune recognition and cancer development, which for example reflect in the much higher mutational loads and immune infiltrate in Melanoma compared to Lung cancer [9].

## 1 Methods

We sought to assess whether certain mutagenic processes result in somatic alterations that increase the similarity of the mutated human exome with selected pathogens. Accordingly, we first defined a pairwise similarity metric among DNA sequences of different length and evaluated the similarity between pathogens and the normal human exome. Second, we simulated mutations resulting from different mutagenic processes at different mutation rates acting on the human exome and evaluated the consequent change in similarity of the mutated human exome with respect to the pathogen exomes. Third, we investigated the resiliency of exomes (human exome and model organism exomes) in terms of maintained functionality of the resulting amino-acids and compared the sequences of amino acids of the normal and mutated exomes.

### Data and computing resources

We obtained the human normal exome from GRCh38 http://www.ensembl.org/Homo_sapiens/Info/Index.

We considered the following list of model organisms: Mus Musculus (Mouse), Saccharomyces Cerevisiae (Yeast), Felis Catus (Cat), Drosophila Melanogaster (Fruitfly), Caenorhabditis Elegans (Nematode), Xenopus, Danio Rerio (Zebrafish), Cavia Porcellus (Pig), Anolis carolinensis (Anolis). Exomes from these organisms were obtained from http://uswest.ensembl.org/biomart/martview/.

We considered the following list of viral pathogens: Cytomegalovirus (CMV), Dengue virus, Ebola virus, Epstein-Barr virus (EBV), Human Herpesvirus 6 (HHV), Human Papillomavirus (HPV), Measles virus, Yellow Fever virus. DNA sequences from these pathogens were obtained from http://www.ncbi.nlm.nih.gov/.

We considered simulations of mutational signatures resulting from ultra-violet (UV) light (specific to Melanoma), smoking (specific to Non Small Cell Lung Cancer (NSCLC)), and APOBEC activation (specific to Bladder cancer). These simulations were based on the data from [8, Supplementary information, Table S2] restricted to the set of patients with Melanoma cancer, NSCLC, and Bladder cancer.

For simulations we used Python 2.7.6 (libraries random, numpy, and scipy.stats) and ran programs on a shared server with 8 CPUs and 128GB memory.

## 2 Results

### 2.1 Pathogen DNA vs. human exome and MHC mechanism

To quantify the similarity between a pathogen DNA, denoted by *x*, and the human exome, denoted by *y*, we considered the following similarity score. For a given integer *ℓ* ≥ 1, the similarity score, denoted by *s*_*ℓ*_(*x*, *y*), corresponds to the relative proportion of length-*ℓ* strings in the pathogen DNA that also appear in the human exome at least once, that is

$$s_{\ell }\left(x,y\right)\overset{\text{def}}{=}\frac{1}{L-\ell +1}\sum_{i=1}^{L-\ell +1}z_{i}$$

where

$$z_{i}\overset{\text{def}}{=}\{\begin{array}{cc} 1 & \text{if}\;x_{i}^{i+\ell -1}≺y\\ 0 & \text{otherwise.} \end{array}$$

Here *L* denotes the length of the pathogen DNA, $x_{i}^{i+\ell -1}\overset{\text{def}}{=}x_{i},x_{i+1},\ldots ,x_{i+\ell -1}$ denotes the pathogen DNA substring starting at position *i* and ending at position *i* + *ℓ* − 1, and “≺” denotes string inclusion. In particular, *s*_*ℓ*_(*x*, *y*) = 1 corresponds to the case where all length-*ℓ* strings in the pathogen DNA also appear in the human exome and *s*_*ℓ*_(*x*, *y*) = 0 corresponds to the case where the pathogen DNA and the human exome have no length-*ℓ* string in common. Observe that *s*_*ℓ*_(*x*, *y*) can be interpreted as the probability that a randomly and uniformly picked length-*ℓ* string in the pathogen DNA also appears in the human exome. Accordingly, we often refer to *s*_*ℓ*_(*x*, *y*) as the matching probability. Finally, notice that *s*_*ℓ*_(*x*, *y*) does not count multiplicity, *i.e*., strings that appear only once in the human exome and strings that appear multiple times in the human exome are note distinguished.

**Fig 1:**
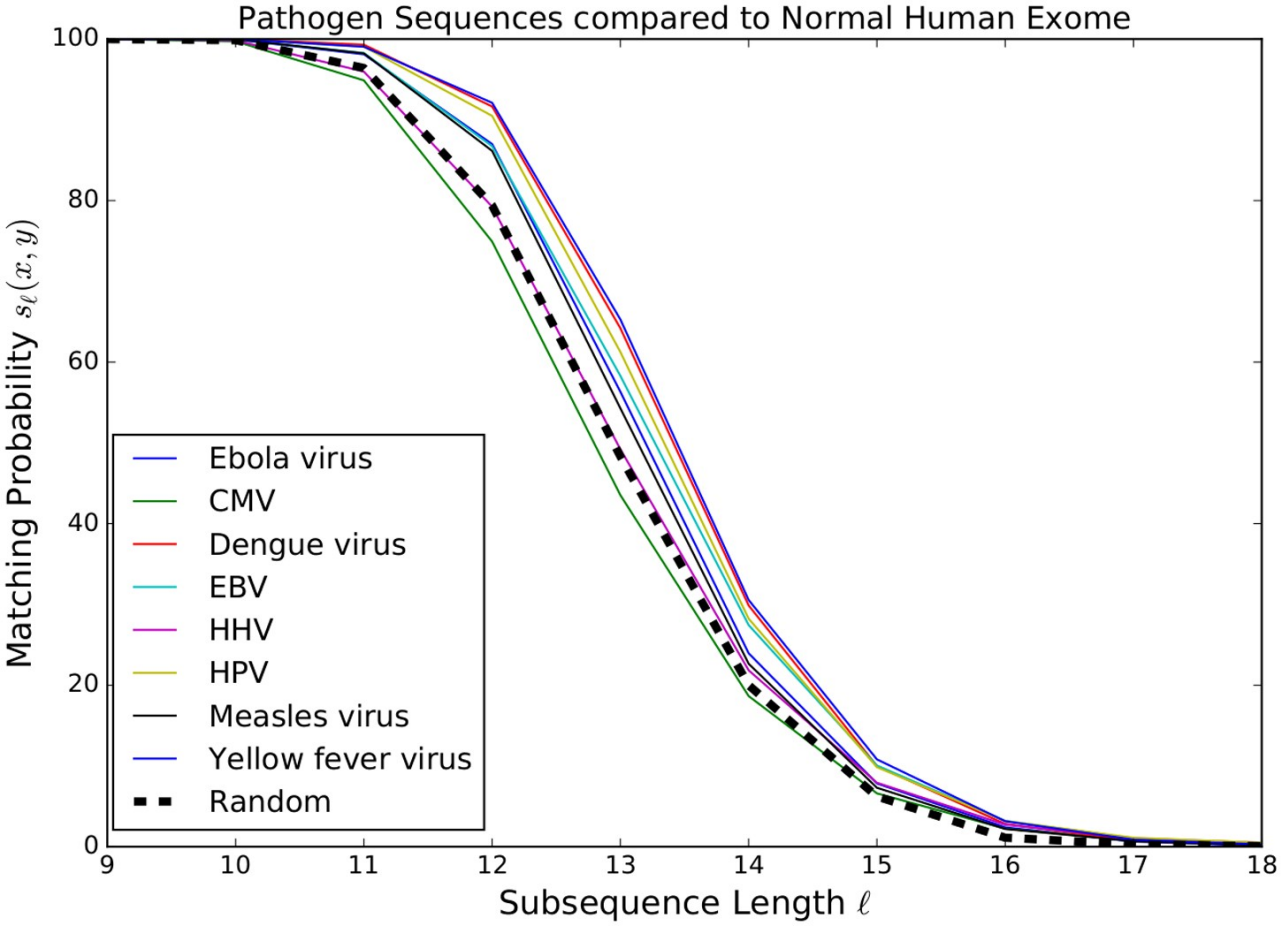
Each curve represents the matching probability (similarity score) *s*_*ℓ*_(*x*, *y*) between a pathogen DNA *x* and the human exome *y*, as a function of the subsequence length *ℓ*. The “Random” curve refers to the average score of a randomly and uniformly generated “pathogen” DNA sequence.

In Fig 1, each curve represents the matching probability *s*_*ℓ*_(*x*, *y*) for a specific pathogen DNA *x* and the normal human exome *y*, for *ℓ* ∈ {9, 10, …, 18}. To benchmark these scores we also considered the matching probability with respect to a randomly and uniformly generated “pathogen” sequence, where each nucleotide is equally likely to occur. The average matching probability with respect to such a sequence is represented by the “Random” curve in Fig 1 and turns out to be independent of its length *L*. This curve is indistinguishable from the 95% confidence interval corresponding to a randomly generated sequence. Supporting material for Fig 1 is deferred to Section A.1 in the Appendix. We make the following observations:

- For all pathogens the similarity score is equal to one for *ℓ* ≤ 10, that is length *ℓ* ≤ 10 subsequences of the pathogen DNAs all appear in the human exome as well.
- The similarity scores are non-zero for all pathogens up to length *ℓ* = 20. At *ℓ* = 21 the similarity scores is zero for the Ebola virus, the Measles virus, and the Dengue virus.
- For all *ℓ* ∈ {11, …, 18} the similarity score for pathogen DNAs is higher than for a random sequence, except for CMV (*ℓ* ∈ {15, …, 18}) and for HHV (*ℓ* ∈ {13, …, 18}).
- From *ℓ* = 10 there is a steep decrease in the similarity scores, down to less than 15% for *ℓ* = 15. A closer look at the data (see Tables in the Appendix A.1) reveals that, for all pathogens, the sharpest relative drop of the similarity score occurs from *ℓ* = 12 to *ℓ* = 13 or from *ℓ* = 13 to *ℓ* = 14.
- The differences in score across pathogens is maximal at *ℓ* ∈ {12, 13}.

These in-silico observations are in line with the concept that 4 – 5 amino acids are enough for the presentation machinery in terms of both diversity of possible sequences (20^4^ – 20^5^) and differentiation of self from foreign sequences in the MHC machinery. Namely, this length is strikingly similar to the length of peptides studied in the signature determined by [11].

### 2.2 Impact of somatic mutations on pathogen DNA and human exome similarity score

To assess the impact of somatic mutations on pathogen DNA and human exome similarity score and identify the roles of mutation distribution and mutation rate we proceeded as follows:

- Normal exome vs. cancer exome: we investigated whether cancer somatic mutations render pathogen and human exome more similar, and whether random mutations alone, with uniform distribution across mutations, would produce the same results as (typically non-uniform) cancer-dependent mutations, at the same mutation rate.
- Impact of mutation rate: we investigated whether a higher mutation rate renders pathogen DNA and human exome more similar.

Central to our investigation is a notion of cancer channel described next.

#### Cancer channel

We simulated the changes induced to the normal exome by cancer specific mutagens in a probabilistic way. The cancer exomes were generated from the normal exome by using cancer-dependent mixtures of mutational signatures with empirical weights derived from data in [8]. Note that even if a cancer typically exhibits a dominant mutational signature, the simulated mutagenic process results in a more realistic combination of such signatures. The similarity scores of the normal exome and cancer exome were then computed for each pathogen. To formalize our analysis, we used concepts from information theory, in particular related to communications over a noisy channel. To a given cancer and mutation rate we associated a transformation, referred to as “cancer channel,” which mimics the typical effects of the mutagenic process that are specific to the cancer at the given mutation rate. Analogously to a communication channel that alterates a transmitted message because of noise (see, *e.g*., [18]), a cancer channel alterates a DNA sequence because of somatic mutations. Given a particular cancer *c* and a mutation rate *ρ* the cancer channel assigns to each nucleotide *α* the probability $P_{c,\rho }\left(\beta |\alpha \right)$ of being mutated into nucleotide *β*. This probability was derived using data from [8, Supplementary information, Table S2] (see Appendix A.2 in this paper).

**Fig 2:**
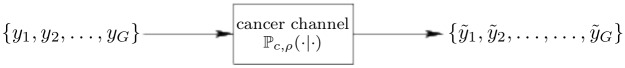
Effects of cancer specific mutations on a normal exome modeled as a cancer channel. Cancer exome $\tilde{y}=\left\{{\tilde{y}}_{1},{\tilde{y}}_{2},\ldots ,{\tilde{y}}_{G}\right\}$ is obtained from normal exome *y* = {*y*_1_, *y*_2_, …, *y*_*G*_} through a cancer specific probabilistic transformation $P_{c,\rho }\left(\beta |\alpha \right)$ which assigns to each nucleotide *α* the probability of being mutated to nucleotide *β*. This transformation depends on both the mutation distribution specific to cancer *c* and the mutation rate *ρ*.

To obtain a cancer exome $\tilde{y}$ we “passed” the normal human exome *y* through cancer channel $P_{c,\rho }\left(\cdot |\cdot \right)$ as shown in Fig 2. Specifically, the cancer exome $\tilde{y}$ was generated from *y* so that the probability to obtain $\tilde{y}=\left\{{\tilde{y}}_{1},{\tilde{y}}_{2},\ldots ,{\tilde{y}}_{G}\right\}$ from normal exome *y* = {*y*_1_, *y*_2_, …, *y*_*G*_} was given by

$$\prod_{i=1}^{G}P_{c,\rho }\left({\tilde{y}}_{i}|y_{i}\right).$$

#### Normal vs. cancer specific and random mutations

For given pathogen *x*, cancer *c*, and mutation rate *ρ* we performed two tests. In Test 1, we evaluated the statistical significance of the effect of cancer somatic mutations in making human exome more similar to pathogen DNA sequences. In Test 2, we compared cancer somatic mutations and random mutations in making the human exome more similar to pathogen DNA sequences. Both tests were peformed for *ρ*-values of 0.0005, 0.001, and 0.01. The lowest mutation rate was chosen to be 0.0005 as it represents a good compromise between biological and statistical relevance. It lies in the upper range of the mutation rates observed in actual cancer samples [8] and in the lower range for statistical relevance—see next subsection.

**Test 1:** For each *ℓ* ∈ {10, 11, …, 18} we independently generated 1000 cancer exomes $\left\{\tilde{y}\right\}$ from the normal human exome *y* and computed the corresponding similarity scores $\left\{s_{\ell }\left(x,\tilde{y}\right)\right\}$. *P*-values were computed for comparing the mean of $\left\{s_{\ell }\left(x,\tilde{y}\right)\right\}$ against *s*_*ℓ*_(*x*, *y*) using a one-sided t-test with a null hypothesis that the true mean of $s_{\ell }\left(x,\tilde{y}\right)$ is no larger than *s*_*ℓ*_(*x*, *y*).

**Test 2:** We replaced the cancer channel by a “random channel” which produced mutations at the same rate but in a uniform (1/3, 1/3, 1/3) manner. For each *ℓ* ∈ {10, 11, …, 18} we independently generated 1000 exomes $\left\{\hat{y}\right\}$ by passing the normal human exome *y* through the random channel and computed the corresponding similarity scores $\left\{s_{\ell }\left(x,\hat{y}\right)\right\}$. *P*-values were computed for comparing the mean of $\left\{s_{\ell }\left(x,\hat{y}\right)\right\}$ against the mean of $\left\{s_{\ell }\left(x,\tilde{y}\right)\right\}$ (obtained in Test 1) using a two-sample one-sided t-test with a null hypothesis that the true mean of $s_{\ell }\left(x,\tilde{y}\right)$ is no larger than the true mean of $s_{\ell }\left(x,\hat{y}\right)$—note that directly computing the true mean of $s_{\ell }\left(x,\tilde{y}\right)$ over $\tilde{y}$ is impossible as it amounts to computing a sum over all $\approx 4^{10^{7}}$ possible cancer exomes, and similarly for the mean of $s_{\ell }\left(x,\hat{y}\right)$.

**Fig 3:**
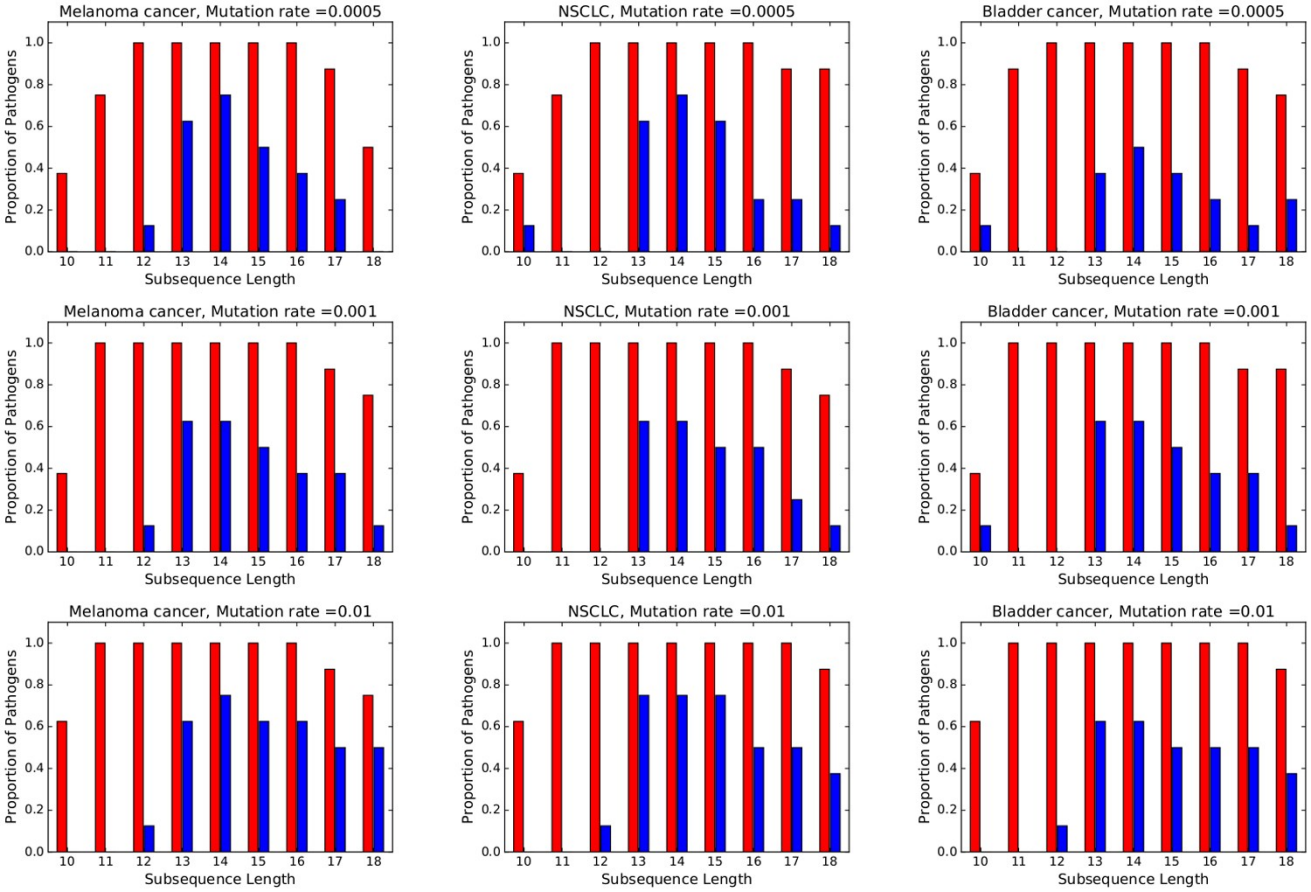
The height of the red bars represents the proportions of pathogen DNA that are more similar to cancer exomes than to normal exome (one-sample t-test results with *p*-value ≤ 0.01). The height of the blue bars represents the proportion of pathogens whose DNA are more similar to cancer exomes than to exomes with equal mutation rate but uniformly distributed mutations (two-sample one-sided t-test *p*-value ≤ 0.01).

In Fig 3, each histogram refers to a particular cancer and mutation rate. Red bars refer to Test 1 and blue bars refer to Test 2. Bar height represents, for any given subsequence length *ℓ* ∈ {10, 11, …, 18}, the proportion of pathogens (out of the 8 considered in this paper) for which the *p*-value is ≤ 0.01. Related data can be found in the tables of the Appendices A.3, A.4, and A.5 for *ρ* = 0.0005, *ρ* = 0.001, and *ρ* = 0.01, respectively. In these tables, the second column refers to *s*_*ℓ*_(*x*, *y*), the third column gives a 95% confidence interval for $s_{\ell }\left(x,\tilde{y}\right)$, the fourth column gives the *p*-value for Test 1 and the fifth column gives the *p*-value for Test 2. We make the following observations:

- Referring to Test 1 (red bars in Fig 3), all three mutagenic processes render the human exome more similar to all pathogen DNA sequences at all *ρ* ∈ {0.0005, 0.001, 0.01} and *ℓ* ∈ {12, …, 16}. For *ℓ* ≤ 11 or *ℓ* ≥ 17 the effect of the mutagenic processes on the similarity scores are less conclusive. This suggests that the increase of similarity is particularly relevant in the range of peptide sizes (4 – 5 amino-acids) that are relevant for epitope presentation in the human MHC presentation. Note, however, that the changes in similarity are small, typically ≪ 1% (see tables in Sections A.3-A.5, Columns 2, 3).
- Whether the above change of similarity is due to the specificity of the mutation distribution or random mutations trigger the same effect depends on the pathogen, the length, and the mutation rate. For instance, for Melanoma at *ℓ* = 13 the change in similarity due to cancer specific mutations is more pronounced for 5 out of the 8 pathogens, for *ρ* ∈ {0.0005, 0.001, 0.01}. By contrast, for all mutagenic processes there appears to be no statistical difference at length 11.

#### Impact of mutation rate

**Fig 4:**
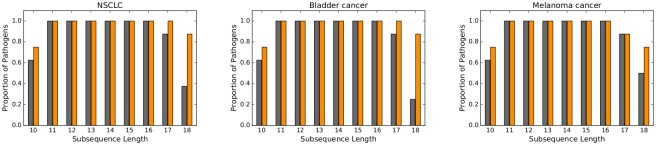
The height of the grey bars represents the proportion of pathogen DNA for which increasing mutation rate from 0.0005 to 0.001 results in an increased similarity with the cancer exome (*p*-value≤ 0.01). Orange bars refer to the same proportions but when mutation rate increases from 0.001 to 0.01.

To assess the impact of mutational rate on the similarity between pathogen DNA and human exome, for any given mutagenic process, pathogen DNA, and length we proceeded as follows. We first generated 1000 cancer exomes at mutation rate *ρ* = 0.0005 and 1000 cancer exomes at mutation rate 0.001. Second, we computed the similarity scores of the two sets of cancer exomes relative to the pathogen DNA. *P*-values were computed for comparing the means of the two sets of similarity scores using a two-sample one-sided t-test with a null hypothesis that the true mean of the similarity scores at the lowest rate (*ρ* = 0.0005) is no larger than the true mean of the similarity scores at the higher rate (*ρ* = 0.001). We then repeated the experiment for *ρ* = 0.001 vs. *ρ* = 0.01. In Fig 4, the histograms represent the proportion of pathogens for which the *p*-value is ≤ 0.01—grey bars refers to the 0.0005 v.s. 0.001 experiment and the orange bars refer to the 0.001 v.s. 0.01 experiment. We obtain the following result:

- For all combinations of mutagenic processes and pathogens, and for all *ℓ* ∈ {11, …, 16}, a higher mutation rate results in higher similarity score. For *ℓ* ∈ {9, 10, 17, 18} results are inconclusive.

### 2.3 Resiliency of exomes with respect to mutagenic processes

**Fig 5:**
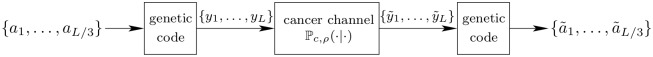
Exome {*y*_1_, *y*_2_, …}, which gives amino acid sequence {*a*_1_, *a*_2_, …}, undergoes specific somatic mutations through transformation $P_{c,\rho }\left(\cdot |\cdot \right)$ and results in $\left\{{\tilde{y}}_{1},{\tilde{y}}_{2},\ldots \right\}$ which, in turn, gives amino acid sequence $\left\{{\tilde{a}}_{1},{\tilde{a}}_{2},\ldots \right\}$.

In order to compare the resiliency of the model organism exomes with respect to mutagenic processes, we evaluated the error correction capabilities of the genetic code (the codon allocation to amino-acids) for each combination of model exome and mutagenic process. Referring to Fig 5, *y* = {*y*_1_, …, *y*_*L*_} represents a DNA sequence whose corresponding sequence of amino acids is {*a*_1_, …, *a*_*L*/3_}. This DNA sequence is then passed through a given cancer channel $P_{c,\rho }\left(\cdot |\cdot \right)$ and results in a cancer sequence $\tilde{y}=\left\{{\tilde{y}}_{1},{\tilde{y}}_{2},\ldots ,{\tilde{y}}_{L}\right\}$ and a corresponding sequence of cancer amino acids $\left\{{\tilde{a}}_{1},{\tilde{a}}_{2},\ldots ,{\tilde{a}}_{L/3}\right\}$. From {*a*_1_, *a*_2_, …, *a*_*L*/3_} and $\left\{{\tilde{a}}_{1},{\tilde{a}}_{2},\ldots ,{\tilde{a}}_{L/3}\right\}$ we computed the relative proportion of amino acids that were affected, that is

$$\begin{array}{c} \frac{\left|\{i:{\tilde{a}}_{i}\neq a_{i}\}\right|}{\left(L/3\right)}. \end{array}$$ (1)

Finally, averaging over all possible realizations of $\tilde{y}$ (and therefore over $\tilde{a}$), we obtained the average error probability

$$\begin{array}{c} P\left(\text{error}|y,c,\rho \right)=\frac{E|\{i:{\tilde{a}}_{i}\neq a_{i}\}|}{\left(L/3\right)}. \end{array}$$ (2)

**Fig 6:**
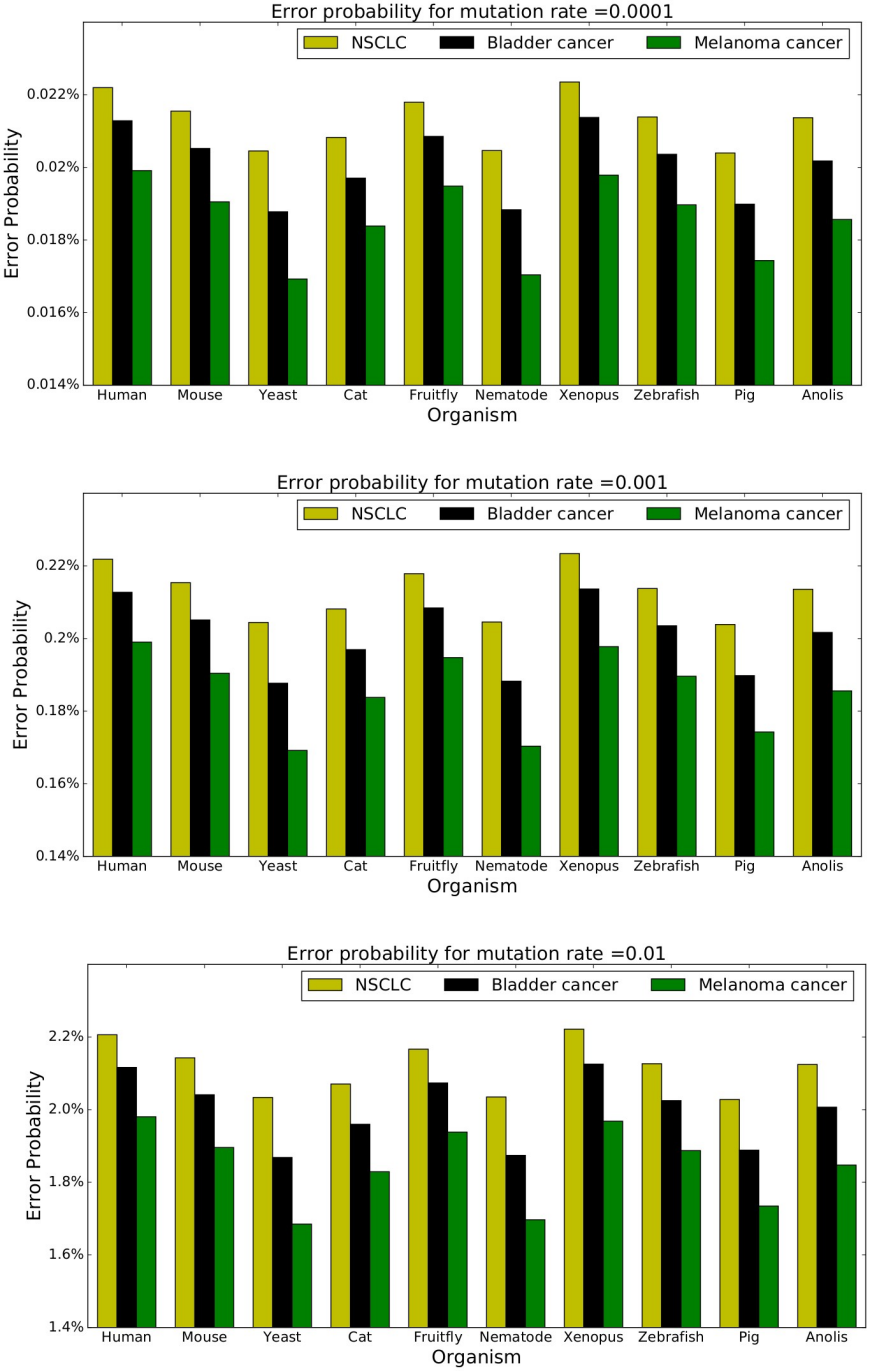
Average proportions of erroneous amino acids after passing exomes through different cancer channels at mutation rater 0.0001, 0.001, and 0.01.

Fig 6 represents P(error|y,c,ρ) for each combination of model organism, cancer mutation process, and mutation rate *ρ* ∈ {0.0001, 0.001, 0.01}. Notice that P(error|y,c,ρ) is not a linear function of *ρ*. Computation details for P(error|y,c,ρ) are deferred to the Appendix A.6. Referring to Fig 6, we obtain the following result:

- Although the proportion of non-synonymous mutations varies across exomes for the three types of mutagenic processes, it is always lowest for melanoma and maximal for lung. Moreover, this ordering holds irrespectively of the intensity of the mutation rate. It should be noted that we evaluated the proportions of non-synonymous mutations for several other organisms as well (including the set of pathogens considered in this paper) and this finding was validated in all cases.

## 3 Discussion

We employed large scale simulations to model the random (across space) effect of stochastic mutagenic processes on the human normal genome. We believe this is a valid approach since the cancer exome available data does suggest that, while at the granular level mutation rates vary, the mutagenic processes in cancers with large number of mutations affect equally all chromosomal regions of the exome [8]. Essentially, we simplify the analysis using this assumption.

Our in-silico results show that, in general, the typical stochastic mutagenic processes encountered in the major cancer indications with abundant neoantigens do appear to shift the peptide distribution of the modified exome universally towards a landscape that appears more similar to pathogenic insult. Specifically, all three mutagenic processes considered induce subtle but robust shifts in the measure by which we characterized the similarity between the normal human exome and pathogen DNA sequences, at mutation rates in the upper range of the mutation rates observed in actual cancer samples (≥ 0.0005). Moreover, the range of peptide lengths where this shift happens aligns with the typical length of peptides presented by the human MHC presentation system, suggesting an increased potential for recognition of these types of somatic mutations by a pathogen-trained host immune system.

We also note that for many combinations of pathogen DNA and mutagenic process cases this increase of similarity cannot be solely attributed to the mutation distribution; randomly and uniformly distributed mutations can cause similar shifts in similarity. By contrast, increasing the mutation rate while keeping the underlying mutation distribution fixed always results in an increased similarity betweeen human exome and pathogen DNA at *ℓ* ∈ {11, …, 16}, which again corresponds to the length of peptides presented by the human presentation system. This suggests that the intensity of the mutational rate is an important parameter that directly affects the similarity between cancer exome and pathogen DNA.

We also observe that the effect of the considered mutagenic processes on the likelihood of observing a non-synonymous alteration is strikingly different across processes but consistent across the species studied in our framework (human and model organisms). Melanoma/UV light alterations are the least likely to result in amino acid functional changes, followed by APOBEC-driven alterations and then by smoking alterations, suggesting different error-correcting capabilities of the living exomes towards this various mutagenic insults. This is an attractive observation from an evolutionary perspective: due to universal exposure to sunlight, organisms likely developed similarly universal intrinsic protection from UV light type of modifications to their exomes via the redundancies in the aminoacid codon allocation. Similarly, APOBEC-activation appears to be a universal innate protection mechanism that allows the cell to induce damaging mutations to foreign organisms, while the mutations resulting from tobacco smoking are less likely to have presented evolutionary pressure. In summary, our in-silico approach reveals two competing mechanisms of tolerance pressure on the major mutagenic processes present in human cancers that modulate the potential immune recognition of alterations at the exome level through pathogen similarity and through functional redundancy; the balance between these mechanisms may significantly contribute to the eventual mutational landscape of advanced cancers.

## A Appendices

### A.1 Data for Fig 1

In Table 1 below we listed the similarity scores *s*_*ℓ*_(*x*, *y*) of each pathogen *x* against the human exome *y*, as a function of the subsequence length *ℓ*.

**Table 1:** Matching score of pathogens against human exome.

*ℓ*	Ebola virus	CMV	Dengue virus	EBV	HHV
9	100.0	100.0	100.0	100.0	100.0
10	99.94	99.71	100.0	99.94	99.83
11	98.11	94.88	99.30	98.26	95.96
12	86.99	74.92	91.65	86.74	79.24
13	56.32	43.52	64.19	58.29	49.20
14	23.94	18.64	29.87	27.43	21.84
15	7.82	6.60	9.98	10.05	7.91
16	2.40	2.23	2.81	3.19	2.73
17	0.62	0.77	0.73	1.02	1.05
18	0.12	0.27	0.19	0.33	0.48
*ℓ*	HPV	Measles virus	Yellow fever virus	Random	
9	100.0	100.0	100.0	100±0.002	
10	99.97	99.97	100.0	100±0.002	
11	99.05	98.23	99.05	96.4±0.002	
12	90.48	86.15	92.10	79.4±0.002	
13	61.24	54.27	65.28	48.3±0.002	
14	28.22	22.64	30.54	20±0.002	
15	9.84	7.29	10.81	6.2±0.002	
16	3.19	2.19	3.15	1.116±0.001	
17	1.10	0.68	0.87	0.28±0.001	
18	0.50	0.19	0.29	0.07±0.001	

The column “Random” refers to a 95% confidence interval for the similarity score between a randomly generated pathogen sequence *X*, where each nucleotide is independently and uniformly selected with probability 1/4, and the normal human exome *y*. To compute this confidence interval we proceeded as follows. The similarity score for a random instance *X* of length *L* is given by

$$s_{\ell }\left(X,y\right)=\frac{1}{L-\ell +1}\sum_{i=1}^{L-\ell +1}Z_{i}$$

where the *Z*_*i*_’s are i.i.d. Bernoulli random variables such that

$$\begin{array}{c} Pr\left(Z_{i}=1\right)=1-Pr\left(Z_{i}=0\right)=\min\left\{1,M_{\ell }/4^{\ell }\right\}\overset{\text{def}}{=}s_{\ell }\left(y\right). \end{array}$$ (3)

Here *M*_*ℓ*_ denotes the number of distinct length-*ℓ* substrings in the human genome and was computed empirically for *ℓ* ∈ {9, 10, …, 15}:

$$M_{9}=262,144\;M_{10}=1,047,125\;M_{11}=4,043,826\;$$

$$M_{12}=13,319,884\;M_{13}=32,427,184\;M_{14}=53,660,993\;M_{15}=66,828,678\;$$

$$\;M_{16}=47,945,739\;M_{17}=49,134,120\;M_{18}=49,785,872$$

Taking expectation over *X* yields

$$E\left(s_{\ell }\left(X,y\right)\right)=EZ_{i}=s_{\ell }\left(y\right).$$

A confidence inteval for *s*_*ℓ*_(*X*, *y*) was computed via Chebyshev’s inequality as follows. We have

$$\begin{array}{c} Pr(|s_{\ell }\left(X,y\right)-s_{\ell }\left(y\right)\left|\geq \varepsilon_{\ell }\right)\leq \frac{\text{Var}(s_{\ell }\left(X,y\right))}{\varepsilon_{\ell }^{2}}=\frac{\text{Var}(\sum_{i=1}^{L-\ell +1}Z_{i})}{{\left(L-\ell +1\right)}^{2}\varepsilon_{\ell }^{2}}. \end{array}$$ (4)

Furthermore,

$$\begin{array}{cc} \text{Var}(\sum_{i=1}^{L-\ell +1}Z_{i}) & =\sum_{i=1}^{L-\ell +1}\text{Var}\left(Z_{i}\right)+2\sum_{i<j}\text{Cov}\left(Z_{i},Z_{j}\right)\\ & =\left(L-\ell +1\right)\text{Var}\left(Z_{1}\right)+2\sum_{i=1}^{L-\ell +1}\sum_{j=i+1}^{i+\ell -1}\text{Cov}\left(Z_{i},Z_{j}\right) \end{array}$$

where for the second equality we used the fact that the *Z*_*i*_’s are identically distributed and that *Z*_*k*_ and *Z*_*j*_ are independent whenever *j* ≥ *k* + *ℓ*. Now

$$\text{Var}\left(Z_{1}\right)=s_{\ell }\left(y\right)\left(1-s_{\ell }\left(y\right)\right)\leq s_{\ell }\left(y\right)$$

and since the *Z*_*i*_’s are binary random variables

$$\begin{array}{cc} \text{Cov}(Z_{i},Z_{j}) & =E\left(Z_{i}Z_{j}\right)-E\left(Z_{i}\right)E\left(Z_{j}\right)\\ & \leq E\left(Z_{i}\right)-E\left(Z_{i}\right)E\left(Z_{j}\right)\\ & =s_{\ell }\left(y\right)\left(1-s_{\ell }\left(y\right)\right)\\ & \leq s_{\ell }\left(y\right). \end{array}$$

Therefore,

$$\begin{array}{c} \text{Var}\left(\sum_{i=1}^{L-\ell +1}Z_{i}\right)\leq s_{\ell }\left(y\right)\left(L-\ell +1\right)\left(1+2\left(\ell -1\right)\right). \end{array}$$ (5)

Finally, from (3), (4), and (5) we get

$$Pr(|s_{\ell }\left(X,y\right)-s_{\ell }\left(y\right)\left|\geq \varepsilon_{\ell }\right)\leq \frac{s_{\ell }\left(y\right)\left(1+2\left(\ell -1\right)\right)}{\left(L-\ell +1\right)\varepsilon_{\ell }^{2}}=\frac{\min\left\{1,M/4^{\ell }\right\}\left(1+2\left(\ell -1\right)\right)}{\left(L-\ell +1\right)\varepsilon_{\ell }^{2}}.$$

To obtain a 95% confidence interval we picked

$$\begin{array}{c} \varepsilon_{\ell }={\left(\frac{\min\left\{1,M/4^{\ell }\right\}\left(1+2\left(\ell -1\right)\right)}{(L-\ell +1)0.05}\right)}^{1/2} \end{array}$$ (6)

which is below 0.002 for all *ℓ* ∈ {9, 10, …, 15} regardless of the pathogen length *L*.

### A.2 Cancer channel

We describe how we obtained cancer channel $P_{c,\rho }\left(\cdot |\cdot \right)$ for a given cancer and mutation rate. For each cancer *c* (Melanoma cancer, NSCLC, Bladder cancer) we considered the set $S_{c}$ of patients in [8, Supplementary information, Table S2] with that cancer. Then, for every mutation *α* → *β* we empirically computed the average proportion of mutations across patients

$$p_{c}\left(\alpha \to \beta \right)\overset{\text{def}}{=}\frac{1}{|S_{c}|}\sum_{i\in S_{c}}p_{c}\left(i,\alpha \to \beta \right)$$

where *p*_*c*_(*i*, *α* → *β*) denotes the proportion of *α* → *β* mutations among all mutations in patient *i* and was computed from [8, Supplementary information, Table S2]. The probability that a nucleotide *α* in the normal exome results in nucleotide *β* in the cancer exome is therefore given by

$$P_{c,\rho }\left(\beta |\alpha \right)=\frac{p_{c}\left(\alpha \to \beta \right)\rho }{p(\alpha )}$$

for *β* ≠ *α* and

$$P_{c,\rho }\left(\alpha |\alpha \right)=1-\frac{\rho }{p(\alpha )}\sum_{\beta \neq \alpha }p_{c}\left(\alpha \to \beta \right).$$

The parameter *ρ* denotes the overall mutation rate and *p*(*α*) denotes the relative number of nucleotide *α* in the exome and was computed from [8, Supplementary information, Table S2].

**Remark**. *Because in the data from* [8, *Supplementary information, Table S2*] *complementary mutations were counted under the same category (e.g., a change from cytosine to tyamine would be treated the same as a change from guanine to adenine), mutation types were considered in pairs. Since the relative proportions of complementary pairs were not given inf, we made the assumption that they were equal. Hence, in the above expression p*_*c*_(*i*, *α* → *β*, *i*) *actually corresponds to*

$$p_{c}\left(i,\alpha \to \beta ,i\right)/2+p_{c}\left(i,\alpha^{\prime }\to \beta^{\prime },i\right)/2$$

*where* (*α*′, *β*′) *is the complementary pair of* (*α*, *β*).

The second column in Tables 2–9 represents *s*_*ℓ*_(*x*, *y*) as a function of *ℓ*. The third column represents a 95% confidence interval for $s_{\ell }\left(x,\tilde{y}\right)$ obtained through a standard application of the central limit theorem. This confidence interval is given by

$$[s_{\ell }\left(x\right)\pm \frac{1.96\sigma }{\sqrt{1000}}],$$

where *s*_*ℓ*_(*x*) denotes the average of $s_{\ell }\left(x,\tilde{y}\right)$ over the 1000 independent trials ${\left\{{\tilde{y}}_{i}\right\}}_{i=1}^{1000}$ and where *σ* denotes the empirical standard deviation of $s_{\ell }\left(x,\tilde{y}\right)$. The fourth column in the tables of Sections A.3-A.5 gives the *p*-value for Test 1 and the fifth column gives the *p*-value for Test 2.

**Table 2:** Ebola virus indication.

*ℓ*	Normal	Lung, *ρ* = 0.0005	P-value	P-value random
9	100.0	99.99±(< 10^−5^)	1.0	0.5
10	99.94	99.94±0.0002	1.0	< 0.01
11	98.11	98.13±0.0011	< 0.01	0.9999
12	86.99	87.08±0.0022	< 0.01	0.8762
13	56.32	56.45±0.0032	< 0.01	< 0.01
14	23.94	24.03±0.0027	< 0.01	< 0.01
15	7.82	7.86±0.0018	< 0.01	< 0.01
16	2.40	2.41±0.0010	< 0.01	< 0.01
17	0.62	0.63±0.0005	< 0.01	< 0.01
18	0.12	0.12±0.0002	< 0.01	0.4808
*ℓ*	Normal	Bladder, *ρ* = 0.0005	P-value	P-value random
9	100.0	99.99±(< 10^−5^)	1.0	0.5
10	99.94	99.94±0.0002	1.0	< 0.01
11	98.11	98.14±0.0012	< 0.01	0.9872
12	86.99	87.08±0.0022	< 0.01	0.8483
13	56.32	56.45±0.0031	< 0.01	< 0.01
14	23.94	24.02±0.0027	< 0.01	< 0.01
15	7.82	7.85±0.0017	< 0.01	< 0.01
16	2.40	2.41±0.0010	< 0.01	< 0.01
17	0.62	0.63±0.0005	< 0.01	0.0831
18	0.12	0.12±0.0003	< 0.01	0.5661
*ℓ*	Normal	Mela, *ρ* = 0.0005	P-value	P-value random
9	100.0	99.99±(< 10^−5^)	1.0	0.5
10	99.94	99.94±0.0002	1.0	0.0706
11	98.11	98.13±0.0010	< 0.01	1.0
12	86.99	87.07±0.0021	< 0.01	0.9999
13	56.32	56.46±0.0032	< 0.01	< 0.01
14	23.94	24.03±0.0027	< 0.01	< 0.01
15	7.82	7.86±0.0018	< 0.01	< 0.01
16	2.40	2.41±0.0010	< 0.01	< 0.01
17	0.62	0.63±0.0005	< 0.01	< 0.01
18	0.12	0.12±0.0003	< 0.01	0.5378

**Table 3:** CMV indication.

*ℓ*	Normal	Lung, *ρ* = 0.0005	P-value	P-value random
9	100.0	99.99±(< 10^−5^)	1.0	0.8501
10	99.71	99.72±0.0001	< 0.01	0.9985
11	94.88	94.94±0.0005	< 0.01	1.0
12	74.92	75.05±0.0008	< 0.01	1.0
13	43.52	43.63±0.0008	< 0.01	1.0
14	18.64	18.70±0.0006	< 0.01	1.0
15	6.60	6.62±0.0004	< 0.01	1.0
16	2.23	2.23±0.0002	< 0.01	0.9999
17	0.77	0.77±0.0001	< 0.01	0.9994
18	0.27	0.27±0.0001	< 0.01	0.9999
*ℓ*	Normal	Bladder, *ρ* = 0.0005	P-value	P-value random
9	100.0	99.99±(< 10^−5^)	1.0	0.7875
10	99.71	99.72±0.0001	< 0.01	0.0189
11	94.88	94.95±0.0005	< 0.01	0.9999
12	74.92	75.07±0.0008	< 0.01	1.0
13	43.52	43.64±0.0009	< 0.01	1.0
14	18.64	18.70±0.0007	< 0.01	0.9999
15	6.60	6.62±0.0004	< 0.01	0.9999
16	2.23	2.24±0.0003	< 0.01	0.9760
17	0.77	0.77±0.0001	< 0.01	0.8426
18	0.27	0.27±0.0001	< 0.01	0.4867
*ℓ*	Normal	Mela, *ρ* = 0.0005	P-value	P-value random
9	100.0	99.99±(< 10^−5^)	1.0	0.1131
10	99.71	99.72±0.0001	< 0.01	0.9999
11	94.88	94.94±0.0005	< 0.01	1.0
12	74.92	75.04±0.0008	< 0.01	1.0
13	43.52	43.62±0.0008	< 0.01	1.0
14	18.64	18.69±0.0007	< 0.01	1.0
15	6.60	6.61±0.0004	< 0.01	1.0
16	2.23	2.23±0.0002	< 0.01	1.0
17	0.77	0.77±0.0001	< 0.01	0.9999
18	0.27	0.27±0.0001	< 0.01	0.7213

**Table 4:** Dengue virus indication.

*ℓ*	Normal	Lung, *ρ* = 0.0005	P-value	P-value random
9	100.0	99.99±(< 10^−5^)	1.0	0.5
10	100.0	99.98±(< 10^−5^)	1.0	0.9714
11	99.30	99.29±0.0010	0.9943	0.9994
12	91.65	91.69±0.0026	< 0.01	0.7855
13	64.19	64.29±0.0041	< 0.01	0.1778
14	29.87	29.94±0.0042	< 0.01	< 0.01
15	9.98	10.01±0.0028	< 0.01	0.0106
16	2.81	2.82±0.0016	< 0.01	0.0929
17	0.73	0.74±0.0008	< 0.01	0.2427
18	0.19	0.19±0.0004	< 0.01	0.9153
*ℓ*	Normal	Bladder, *ρ* = 0.0005	P-value	P-value random
9	100.0	99.99±(< 10^−5^)	1.0	0.5
10	100.0	99.99±(< 10^−5^)	1.0	0.8042
11	99.30	99.30±0.0010	< 0.01	0.3967
12	91.65	91.69±0.0026	< 0.01	0.6794
13	64.19	64.29±0.0042	< 0.01	0.1061
14	29.87	29.93±0.0038	< 0.01	0.0510
15	9.98	10.01±0.0026	< 0.01	0.1582
16	2.81	2.82±0.0016	< 0.01	0.1172
17	0.73	0.74±0.0008	< 0.01	0.1540
18	0.19	0.19±0.0004	0.0110	0.9427
*ℓ*	Normal	Mela, *ρ* = 0.0005	P-value	P-value random
9	100.0	99.99±(< 10^−5^)	1.0	0.5
10	100.0	99.99±(< 10^−5^)	1.0	0.5910
11	99.30	99.29±0.0010	0.9987	0.9998
12	91.65	91.69±0.0027	< 0.01	0.9877
13	64.19	64.29±0.0043	< 0.01	< 0.01
14	29.87	29.94±0.0041	< 0.01	< 0.01
15	9.98	10.02±0.0027	< 0.01	< 0.01
16	2.81	2.82±0.0015	< 0.01	0.0267
17	0.73	0.74±0.0009	< 0.01	0.2552
18	0.19	0.19±0.0005	0.0911	0.9770

**Table 5:** EBV indication.

*ℓ*	Normal	Lung, *ρ* = 0.0005	P-value	P-value random
9	100.0	99.99±(< 10^−5^)	1.0	0.9525
10	99.94	99.94±(< 10^−5^)	< 0.01	0.9863
11	98.26	98.29±0.0004	< 0.01	1.0
12	86.74	86.82±0.0008	< 0.01	1.0
13	58.29	58.39±0.0011	< 0.01	1.0
14	27.43	27.49±0.0010	< 0.01	1.0
15	10.05	10.07±0.0008	< 0.01	1.0
16	3.19	3.20±0.0005	< 0.01	0.9999
17	1.02	1.02±0.0003	< 0.01	0.9999
18	0.33	0.33±0.0002	< 0.01	0.9997
*ℓ*	Normal	Bladder, *ρ* = 0.0005	P-value	P-value random
9	100.0	99.99±(< 10^−5^)	1.0	0.7931
10	99.94	99.94±(< 10^−5^)	< 0.01	0.2122
11	98.26	98.29±0.0004	< 0.01	0.9999
12	86.74	86.83±0.0009	< 0.01	1.0
13	58.29	58.40±0.0011	< 0.01	1.0
14	27.43	27.49±0.0010	< 0.01	1.0
15	10.05	10.07±0.0008	< 0.01	1.0
16	3.19	3.20±0.0006	< 0.01	0.9999
17	1.02	1.02±0.0003	< 0.01	0.9999
18	0.33	0.33±0.0002	< 0.01	0.9997
*ℓ*	Normal	Mela, *ρ* = 0.0005	P-value	P-value random
9	100.0	99.99±(< 10^−5^)	1.0	0.2818
10	99.94	99.94±(< 10^−5^)	< 0.01	0.9409
11	98.26	98.28±0.0004	< 0.01	1.0
12	86.74	86.82±0.0008	< 0.01	1.0
13	58.29	58.38±0.0011	< 0.01	1.0
14	27.43	27.48±0.0010	< 0.01	1.0
15	10.05	10.06±0.0008	< 0.01	1.0
16	3.19	3.20±0.0005	< 0.01	1.0
17	1.02	1.02±0.0003	< 0.01	1.0
18	0.33	0.33±0.0002	0.8882	0.9999

**Table 6:** HHV indication.

*ℓ*	Normal	Lung, *ρ* = 0.0005	P-value	P-value random
9	100.0	99.99±(< 10^−5^)	1.0	0.5
10	99.83	99.83±0.0001	< 0.01	0.9998
11	95.96	96.02±0.0006	< 0.01	1.0
12	79.24	79.36±0.0013	< 0.01	1.0
13	49.20	49.32±0.0015	< 0.01	< 0.01
14	21.84	21.91±0.0011	< 0.01	< 0.01
15	7.91	7.94±0.0007	< 0.01	< 0.01
16	2.73	2.74±0.0004	< 0.01	< 0.01
17	1.05	1.05±0.0003	< 0.01	< 0.01
18	0.48	0.48±0.0003	< 0.01	< 0.01
*ℓ*	Normal	Bladder, *ρ* = 0.0005	P-value	P-value random
9	100.0	99.99±(< 10^−5^)	1.0	0.0510
10	99.83	99.83±0.0001	< 0.01	0.9294
11	95.96	96.02±0.0006	< 0.01	0.9999
12	79.24	79.37±0.0014	< 0.01	0.9773
13	49.20	49.32±0.0015	< 0.01	< 0.01
14	21.84	21.91±0.0012	< 0.01	< 0.01
15	7.91	7.94±0.0007	< 0.01	< 0.01
16	2.73	2.74±0.0005	< 0.01	< 0.01
17	1.05	1.05±0.0003	< 0.01	< 0.01
18	0.48	0.48±0.0002	< 0.01	< 0.01
*ℓ*	Normal	Mela, *ρ* = 0.0005	P-value	P-value random
9	100.0	99.99±(< 10^−5^)	1.0	0.7979
10	99.83	99.83±0.0001	< 0.01	0.9999
11	95.96	96.01±0.0006	< 0.01	1.0
12	79.24	79.36±0.0015	< 0.01	1.0
13	49.20	49.32±0.0015	< 0.01	< 0.01
14	21.84	21.91±0.0013	< 0.01	< 0.01
15	7.91	7.94±0.0007	< 0.01	< 0.01
16	2.73	2.74±0.0005	< 0.01	< 0.01
17	1.05	1.05±0.0003	< 0.01	< 0.01
18	0.48	0.48±0.0004	0.0614	0.0148

**Table 7:** HPV indication.

*ℓ*	Normal	Lung, *ρ* = 0.0005	P-value	P-value random
9	100.0	99.98±0.0	1.0	nan
10	99.97	99.96±0.0002	1.0	0.3552
11	99.05	99.04±0.0012	0.9761	0.9939
12	90.48	90.54±0.0032	< 0.01	0.0528
13	61.24	61.37±0.0048	< 0.01	< 0.01
14	28.22	28.30±0.0042	< 0.01	< 0.01
15	9.84	9.88±0.0030	< 0.01	< 0.01
16	3.19	3.20±0.0017	< 0.01	0.0111
17	1.10	1.10±0.0010	< 0.01	0.4128
18	0.50	0.50±0.0006	< 0.01	0.5864
*ℓ*	Normal	Bladder, *ρ* = 0.0005	P-value	P-value random
9	100.0	99.98±0.0	1.0	nan
10	99.97	99.96±0.0002	1.0	0.8341
11	99.05	99.04±0.0011	0.9898	0.9969
12	90.48	90.54±0.0030	< 0.01	0.5926
13	61.24	61.36±0.0049	< 0.01	< 0.01
14	28.22	28.29±0.0040	< 0.01	< 0.01
15	9.84	9.88±0.0029	< 0.01	< 0.01
16	3.19	3.20±0.0017	< 0.01	0.0160
17	1.10	1.10±0.0010	< 0.01	0.8741
18	0.50	0.50±0.0005	< 0.01	0.7670
*ℓ*	Normal	Mela, *ρ* = 0.0005	P-value	P-value random
9	100.0	99.98±0.0	1.0	nan
10	99.97	99.96±0.0003	1.0	0.2953
11	99.05	99.04±0.0011	0.9943	0.9980
12	90.48	90.54±0.0030	< 0.01	< 0.01
13	61.24	61.38±0.0046	< 0.01	< 0.01
14	28.22	28.31±0.0041	< 0.01	< 0.01
15	9.84	9.88±0.0030	< 0.01	< 0.01
16	3.19	3.20±0.0018	< 0.01	< 0.01
17	1.10	1.10±0.0010	< 0.01	0.3923
18	0.50	0.50±0.0006	< 0.01	0.6700

**Table 8:** Measles virus indication.

*ℓ*	Normal	Lung, *ρ* = 0.0005	P-value	P-value random
9	100.0	99.99±(< 10^−5^)	1.0	0.5
10	99.97	99.96±0.0002	1.0	0.9512
11	98.23	98.25±0.0012	< 0.01	0.9931
12	86.15	86.24±0.0025	< 0.01	0.0257
13	54.27	54.40±0.0035	< 0.01	< 0.01
14	22.64	22.72±0.0030	< 0.01	< 0.01
15	7.29	7.32±0.0019	< 0.01	< 0.01
16	2.19	2.20±0.0011	< 0.01	0.0878
17	0.68	0.68±0.0006	0.7775	0.9909
18	0.19	0.19±0.0003	0.1692	0.4103
*ℓ*	Normal	Bladder, *ρ* = 0.0005	P-value	P-value random
9	100.0	99.99±(< 10^−5^)	1.0	0.5
10	99.97	99.96±0.0002	1.0	0.8931
11	98.23	98.25±0.0012	< 0.01	0.9887
12	86.15	86.24±0.0027	< 0.01	0.6929
13	54.27	54.40±0.0034	< 0.01	0.0118
14	22.64	22.72±0.0029	< 0.01	< 0.01
15	7.29	7.32±0.0020	< 0.01	0.3013
16	2.19	2.20±0.0011	< 0.01	0.4939
17	0.68	0.68±0.0006	0.0737	0.8014
18	0.19	0.19±0.0003	0.6188	0.7503
*ℓ*	Normal	Mela, *ρ* = 0.0005	P-value	P-value random
9	100.0	99.99±(< 10^−5^)	1.0	0.5
10	99.97	99.96±0.0002	1.0	0.9656
11	98.23	98.25±0.0012	< 0.01	0.9999
12	86.15	86.24±0.0025	< 0.01	0.5577
13	54.27	54.40±0.0034	< 0.01	< 0.01
14	22.64	22.72±0.0030	< 0.01	< 0.01
15	7.29	7.32±0.0020	< 0.01	0.1858
16	2.19	2.20±0.0011	< 0.01	0.4911
17	0.68	0.68±0.0006	0.2713	0.9212
18	0.19	0.19±0.0003	0.9773	0.9687

**Table 9:** Yellow fever virus indication.

*ℓ*	Normal	Lung, *ρ* = 0.0005	P-value	P-value random
9	100.0	99.99±(< 10^−5^)	1.0	0.5
10	100.0	99.98±0.0001	1.0	0.7992
11	99.05	99.05±0.0011	< 0.01	0.9959
12	92.10	92.16±0.0028	< 0.01	0.7110
13	65.28	65.38±0.0042	< 0.01	< 0.01
14	30.54	30.61±0.0040	< 0.01	< 0.01
15	10.81	10.83±0.0028	< 0.01	< 0.01
16	3.15	3.16±0.0017	< 0.01	0.3423
17	0.87	0.87±0.0009	< 0.01	0.0814
18	0.29	0.29±0.0005	< 0.01	0.1116
*ℓ*	Normal	Bladder, *ρ* = 0.0005	P-value	P-value random
9	100.0	99.99±(< 10^−5^)	1.0	0.5
10	100.0	99.98±0.0001	1.0	0.4066
11	99.05	99.05±0.0011	< 0.01	0.9801
12	92.10	92.16±0.0027	< 0.01	0.7498
13	65.28	65.38±0.0041	< 0.01	0.0445
14	30.54	30.61±0.0042	< 0.01	0.0112
15	10.81	10.83±0.0028	< 0.01	0.2486
16	3.15	3.16±0.0017	< 0.01	0.6824
17	0.87	0.87±0.0009	< 0.01	0.0726
18	0.29	0.29±0.0006	< 0.01	< 0.01
*ℓ*	Normal	Mela, *ρ* = 0.0005	P-value	P-value random
9	100.0	99.99±(< 10^−5^)	1.0	0.5
10	100.0	99.98±0.0001	1.0	0.3586
11	99.05	99.05±0.0011	< 0.01	0.9999
12	92.10	92.16±0.0025	< 0.01	0.9998
13	65.28	65.38±0.0043	< 0.01	0.0127
14	30.54	30.61±0.0040	< 0.01	< 0.01
15	10.81	10.83±0.0028	< 0.01	0.0410
16	3.15	3.16±0.0016	< 0.01	0.2823
17	0.87	0.87±0.0009	< 0.01	0.2685
18	0.29	0.29±0.0005	< 0.01	0.0670

**A.3** *ρ* = 0.0005.

**A.4** *ρ* = 0.001.

Tables 10–17.

**Table 10:** Ebola virus indication.

*ℓ*	Normal	Lung, *ρ* = 0.001	P-value	P-value random
9	100.0	99.99±(< 10^−5^)	1.0	0.5
10	99.94	99.94±0.0003	1.0	0.3328
11	98.11	98.16±0.0015	< 0.01	0.9999
12	86.99	87.16±0.0031	< 0.01	0.2037
13	56.32	56.58±0.0045	< 0.01	< 0.01
14	23.94	24.11±0.0040	< 0.01	< 0.01
15	7.82	7.88±0.0025	< 0.01	< 0.01
16	2.40	2.42±0.0014	< 0.01	< 0.01
17	0.62	0.63±0.0008	< 0.01	< 0.01
18	0.12	0.12±0.0004	< 0.01	0.0393
*ℓ*	Normal	Bladder, *ρ* = 0.001	P-value	P-value random
9	100.0	99.99±(< 10^−5^)	1.0	0.5
10	99.94	99.94±0.0003	0.9999	< 0.01
11	98.11	98.17±0.0015	< 0.01	0.9999
12	86.99	87.16±0.0031	< 0.01	0.4714
13	56.32	56.57±0.0043	< 0.01	< 0.01
14	23.94	24.10±0.0038	< 0.01	< 0.01
15	7.82	7.88±0.0025	< 0.01	< 0.01
16	2.40	2.42±0.0014	< 0.01	< 0.01
17	0.62	0.63±0.0008	< 0.01	< 0.01
18	0.12	0.12±0.0004	< 0.01	0.0521
*ℓ*	Normal	Mela, *ρ* = 0.001	P-value	P-value random
9	100.0	99.99±(< 10^−5^)	1.0	0.5
10	99.94	99.94±0.0002	1.0	0.8304
11	98.11	98.16±0.0015	< 0.01	1.0
12	86.99	87.15±0.0032	< 0.01	0.9989
13	56.32	56.59±0.0045	< 0.01	< 0.01
14	23.94	24.12±0.0039	< 0.01	< 0.01
15	7.82	7.89±0.0025	< 0.01	< 0.01
16	2.40	2.42±0.0014	< 0.01	< 0.01
17	0.62	0.63±0.0007	< 0.01	< 0.01
18	0.12	0.12±0.0004	< 0.01	0.1217

**Table 11:** CMV indication.

*ℓ*	Normal	Lung, *ρ* = 0.001	P-value	P-value random
9	100.0	99.99±(< 10^−5^)	1.0	0.6082
10	99.71	99.73±0.0002	< 0.01	0.9999
11	94.88	95.01±0.0007	< 0.01	1.0
12	74.92	75.18±0.0011	< 0.01	1.0
13	43.52	43.74±0.0012	< 0.01	1.0
14	18.64	18.75±0.0010	< 0.01	1.0
15	6.60	6.63±0.0006	< 0.01	1.0
16	2.23	2.24±0.0003	< 0.01	1.0
17	0.77	0.77±0.0002	< 0.01	0.9999
18	0.27	0.27±0.0001	< 0.01	0.9987
*ℓ*	Normal	Bladder, *ρ* = 0.001	P-value	P-value random
9	100.0	99.99±(< 10^−5^)	1.0	0.5183
10	99.71	99.73±0.0002	< 0.01	0.0255
11	94.88	95.02±0.0007	< 0.01	0.9999
12	74.92	75.21±0.0011	< 0.01	1.0
13	43.52	43.76±0.0012	< 0.01	1.0
14	18.64	18.76±0.0010	< 0.01	1.0
15	6.60	6.64±0.0007	< 0.01	0.9999
16	2.23	2.24±0.0004	< 0.01	0.9999
17	0.77	0.77±0.0002	< 0.01	0.5676
18	0.27	0.27±0.0001	< 0.01	0.3313
*ℓ*	Normal	Mela, *ρ* = 0.001	P-value	P-value random
9	100.0	99.99±(< 10^−5^)	1.0	0.7682
10	99.71	99.73±0.0002	< 0.01	0.9999
11	94.88	95.00±0.0007	< 0.01	1.0
12	74.92	75.16±0.0011	< 0.01	1.0
13	43.52	43.72±0.0012	< 0.01	1.0
14	18.64	18.73±0.0010	< 0.01	1.0
15	6.60	6.63±0.0006	< 0.01	1.0
16	2.23	2.24±0.0004	< 0.01	1.0
17	0.77	0.77±0.0002	< 0.01	0.9999
18	0.27	0.27±0.0001	< 0.01	0.7692

**Table 12:** Dengue virus indication.

*ℓ*	Normal	Lung, *ρ* = 0.001	P-value	P-value random
9	100.0	99.99±(< 10^−5^)	1.0	0.5
10	100.0	99.98±(< 10^−5^)	1.0	0.3914
11	99.30	99.31±0.0014	< 0.01	0.9896
12	91.65	91.75±0.0038	< 0.01	0.9918
13	64.19	64.40±0.0060	< 0.01	< 0.01
14	29.87	30.01±0.0056	< 0.01	< 0.01
15	9.98	10.04±0.0039	< 0.01	< 0.01
16	2.81	2.83±0.0023	< 0.01	< 0.01
17	0.73	0.74±0.0012	< 0.01	0.4424
18	0.19	0.19±0.0005	0.0456	0.9791
*ℓ*	Normal	Bladder, *ρ* = 0.001	P-value	P-value random
9	100.0	99.99±(< 10^−5^)	1.0	0.5
10	100.0	99.98±0.0001	1.0	0.8145
11	99.30	99.31±0.0014	< 0.01	0.9694
12	91.65	91.75±0.0036	< 0.01	0.9986
13	64.19	64.39±0.0059	< 0.01	< 0.01
14	29.87	30.01±0.0057	< 0.01	< 0.01
15	9.98	10.04±0.0039	< 0.01	< 0.01
16	2.81	2.83±0.0021	< 0.01	0.0433
17	0.73	0.74±0.0012	< 0.01	0.5961
18	0.19	0.19±0.0006	< 0.01	0.8638
*ℓ*	Normal	Mela, *ρ* = 0.001	P-value	P-value random
9	100.0	99.99±(< 10^−5^)	1.0	0.5
10	100.0	99.98±(< 10^−5^)	1.0	0.4457
11	99.30	99.30±0.0013	< 0.01	0.9999
12	91.65	91.75±0.0035	< 0.01	0.9999
13	64.19	64.41±0.0061	< 0.01	< 0.01
14	29.87	30.02±0.0055	< 0.01	< 0.01
15	9.98	10.05±0.0037	< 0.01	< 0.01
16	2.81	2.83±0.0023	< 0.01	0.2108
17	0.73	0.74±0.0012	< 0.01	0.3675
18	0.19	0.19±0.0006	< 0.01	0.7893

**Table 13:** EBV indication.

*ℓ*	Normal	Lung, *ρ* = 0.001	P-value	P-value random
9	100.0	99.99±(< 10^−5^)	1.0	0.1418
10	99.94	99.95±0.0001	< 0.01	0.7993
11	98.26	98.31±0.0005	< 0.01	1.0
12	86.74	86.91±0.0012	< 0.01	1.0
13	58.29	58.49±0.0016	< 0.01	1.0
14	27.43	27.54±0.0015	< 0.01	1.0
15	10.05	10.08±0.0012	< 0.01	1.0
16	3.19	3.20±0.0008	< 0.01	1.0
17	1.02	1.03±0.0004	< 0.01	0.9999
18	0.33	0.33±0.0002	< 0.01	0.9999
*ℓ*	Normal	Bladder, *ρ* = 0.001	P-value	P-value random
9	100.0	99.99±(< 10^−5^)	1.0	0.2185
10	99.94	99.95±0.0001	< 0.01	0.1288
11	98.26	98.31±0.0005	< 0.01	0.9999
12	86.74	86.92±0.0012	< 0.01	1.0
13	58.29	58.50±0.0016	< 0.01	1.0
14	27.43	27.55±0.0014	< 0.01	1.0
15	10.05	10.09±0.0012	< 0.01	1.0
16	3.19	3.20±0.0008	< 0.01	1.0
17	1.02	1.03±0.0004	< 0.01	0.9999
18	0.33	0.33±0.0003	< 0.01	0.9999
*ℓ*	Normal	Mela, *ρ* = 0.001	P-value	P-value random
9	100.0	99.99±(< 10^−5^)	1.0	0.0412
10	99.94	99.95±0.0001	< 0.01	0.9878
11	98.26	98.31±0.0005	< 0.01	1.0
12	86.74	86.90±0.0012	< 0.01	1.0
13	58.29	58.47±0.0016	< 0.01	1.0
14	27.43	27.52±0.0014	< 0.01	1.0
15	10.05	10.07±0.0011	< 0.01	1.0
16	3.19	3.20±0.0008	< 0.01	1.0
17	1.02	1.02±0.0004	< 0.01	1.0
18	0.33	0.33±0.0002	0.9238	1.0

**Table 14:** HHV indication.

*ℓ*	Normal	Lung, *ρ* = 0.001	P-value	P-value random
9	100.0	99.99±(< 10^−5^)	1.0	0.2959
10	99.83	99.84±0.0002	< 0.01	1.0
11	95.96	96.07±0.0008	< 0.01	1.0
12	79.24	79.48±0.0018	< 0.01	1.0
13	49.20	49.45±0.0021	< 0.01	< 0.01
14	21.84	21.98±0.0018	< 0.01	< 0.01
15	7.91	7.97±0.0010	< 0.01	< 0.01
16	2.73	2.75±0.0007	< 0.01	< 0.01
17	1.05	1.06±0.0005	< 0.01	< 0.01
18	0.48	0.48±0.0004	< 0.01	< 0.01
*ℓ*	Normal	Bladder, *ρ* = 0.001	P-value	P-value random
9	100.0	99.99±(< 10^−5^)	1.0	0.5000
10	99.83	99.84±0.0002	< 0.01	0.9739
11	95.96	96.08±0.0008	< 0.01	1.0
12	79.24	79.49±0.0018	< 0.01	0.9999
13	49.20	49.45±0.0023	< 0.01	< 0.01
14	21.84	21.98±0.0018	< 0.01	< 0.01
15	7.91	7.97±0.0010	< 0.01	< 0.01
16	2.73	2.75±0.0007	< 0.01	< 0.01
17	1.05	1.06±0.0004	< 0.01	< 0.01
18	0.48	0.48±0.0005	< 0.01	< 0.01
*ℓ*	Normal	Mela, *ρ* = 0.001	P-value	P-value random
9	100.0	99.99±(< 10^−5^)	1.0	0.8155
10	99.83	99.84±0.0002	< 0.01	1.0
11	95.96	96.06±0.0008	< 0.01	1.0
12	79.24	79.47±0.0020	< 0.01	1.0
13	49.20	49.45±0.0022	< 0.01	< 0.01
14	21.84	21.99±0.0017	< 0.01	< 0.01
15	7.91	7.97±0.0011	< 0.01	< 0.01
16	2.73	2.75±0.0007	< 0.01	< 0.01
17	1.05	1.06±0.0004	< 0.01	< 0.01
18	0.48	0.48±0.0005	< 0.01	< 0.01

**Table 15:** HPV indication.

*ℓ*	Normal	Lung, *ρ* = 0.001	P-value	P-value random
9	100.0	99.98±(< 10^−5^)	1.0	0.5
10	99.97	99.96±0.0004	1.0	0.4823
11	99.05	99.05±0.0016	< 0.01	0.9992
12	90.48	90.61±0.0044	< 0.01	0.0447
13	61.24	61.48±0.0068	< 0.01	< 0.01
14	28.22	28.39±0.0062	< 0.01	< 0.01
15	9.84	9.91±0.0042	< 0.01	< 0.01
16	3.19	3.22±0.0024	< 0.01	< 0.01
17	1.10	1.11±0.0015	< 0.01	0.0765
18	0.50	0.50±0.0009	< 0.01	0.3499
*ℓ*	Normal	Bladder, *ρ* = 0.001	P-value	P-value random
9	100.0	99.98±(< 10^−5^)	1.0	0.5
10	99.97	99.96±0.0003	1.0	0.2536
11	99.05	99.06±0.0016	< 0.01	0.9969
12	90.48	90.61±0.0044	< 0.01	0.2479
13	61.24	61.47±0.0067	< 0.01	< 0.01
14	28.22	28.38±0.0061	< 0.01	< 0.01
15	9.84	9.91±0.0040	< 0.01	< 0.01
16	3.19	3.21±0.0025	< 0.01	< 0.01
17	1.10	1.10±0.0014	< 0.01	0.4720
18	0.50	0.50±0.0008	< 0.01	0.8303
*ℓ*	Normal	Mela, *ρ* = 0.001	P-value	P-value random
9	100.0	99.98±(< 10^−5^)	1.0	0.5
10	99.97	99.96±0.0003	1.0	0.4093
11	99.05	99.05±0.0016	< 0.01	0.9998
12	90.48	90.62±0.0043	< 0.01	< 0.01
13	61.24	61.50±0.0069	< 0.01	< 0.01
14	28.22	28.40±0.0059	< 0.01	< 0.01
15	9.84	9.92±0.0040	< 0.01	< 0.01
16	3.19	3.22±0.0025	< 0.01	< 0.01
17	1.10	1.11±0.0014	< 0.01	0.0188
18	0.50	0.50±0.0008	< 0.01	0.3983

**Table 16:** Measles virus indication.

*ℓ*	Normal	Lung, *ρ* = 0.001	P-value	P-value random
9	100.0	99.99±(< 10^−5^)	1.0	0.5
10	99.97	99.97±0.0003	1.0	0.4318
11	98.23	98.28±0.0017	< 0.01	0.9998
12	86.15	86.34±0.0036	< 0.01	0.5411
13	54.27	54.53±0.0050	< 0.01	< 0.01
14	22.64	22.79±0.0043	< 0.01	< 0.01
15	7.29	7.34±0.0028	< 0.01	0.1535
16	2.19	2.20±0.0015	< 0.01	0.3683
17	0.68	0.68±0.0008	0.0104	0.4496
18	0.19	0.19±0.0004	0.9896	0.7992
*ℓ*	Normal	Bladder, *ρ* = 0.001	P-value	P-value random
9	100.0	99.99±(< 10^−5^)	1.0	0.5
10	99.97	99.97±0.0003	1.0	0.3667
11	98.23	98.28±0.0017	< 0.01	0.9969
12	86.15	86.34±0.0035	< 0.01	0.1666
13	54.27	54.52±0.0050	< 0.01	< 0.01
14	22.64	22.78±0.0042	< 0.01	< 0.01
15	7.29	7.34±0.0029	< 0.01	0.9235
16	2.19	2.20±0.0016	< 0.01	0.5732
17	0.68	0.68±0.0009	0.2049	0.7833
18	0.19	0.19±0.0004	0.3444	0.1426
*ℓ*	Normal	Mela, *ρ* = 0.001	P-value	P-value random
9	100.0	99.99±(< 10^−5^)	1.0	0.5
10	99.97	99.97±0.0002	1.0	0.1908
11	98.23	98.27±0.0015	< 0.01	0.9999
12	86.15	86.34±0.0037	< 0.01	0.9581
13	54.27	54.53±0.0047	< 0.01	< 0.01
14	22.64	22.78±0.0041	< 0.01	< 0.01
15	7.29	7.34±0.0027	< 0.01	0.9399
16	2.19	2.20±0.0016	< 0.01	0.2985
17	0.68	0.68±0.0009	0.3218	0.8489
18	0.19	0.19±0.0004	0.8608	0.4713

**Table 17:** Yellow fever virus indication.

*ℓ*	Normal	Lung, *ρ* = 0.001	P-value	P-value random
9	100.0	99.99±(< 10^−5^)	1.0	0.5
10	100.0	99.98±0.0001	1.0	0.9805
11	99.05	99.07±0.0015	< 0.01	0.9999
12	92.10	92.21±0.0038	< 0.01	0.9061
13	65.28	65.48±0.0059	< 0.01	0.6362
14	30.54	30.68±0.0056	< 0.01	0.1009
15	10.81	10.86±0.0040	< 0.01	0.2397
16	3.15	3.17±0.0024	< 0.01	0.7796
17	0.87	0.87±0.0013	< 0.01	0.0412
18	0.29	0.29±0.0007	< 0.01	0.1197
*ℓ*	Normal	Bladder, *ρ* = 0.001	P-value	P-value random
9	100.0	99.99±(< 10^−5^)	1.0	0.5
10	100.0	99.98±0.0001	1.0	0.9826
11	99.05	99.07±0.0015	< 0.01	0.8695
12	92.10	92.21±0.0038	< 0.01	0.9591
13	65.28	65.47±0.0062	< 0.01	0.8768
14	30.54	30.67±0.0058	< 0.01	0.6535
15	10.81	10.85±0.0040	< 0.01	0.3259
16	3.15	3.17±0.0024	< 0.01	0.7424
17	0.87	0.88±0.0013	< 0.01	< 0.01
18	0.29	0.29±0.0008	< 0.01	0.0134
*ℓ*	Normal	Mela, *ρ* = 0.001	P-value	P-value random
9	100.0	99.99±(< 10^−5^)	1.0	0.5
10	100.0	99.98±0.0001	1.0	0.9725
11	99.05	99.06±0.0015	< 0.01	0.9999
12	92.10	92.20±0.0038	< 0.01	0.9993
13	65.28	65.48±0.0059	< 0.01	0.4637
14	30.54	30.68±0.0057	< 0.01	0.1347
15	10.81	10.86±0.0039	< 0.01	0.0967
16	3.15	3.17±0.0023	< 0.01	0.0534
17	0.87	0.87±0.0014	< 0.01	< 0.01
18	0.29	0.29±0.0007	< 0.01	0.0580

**A.5** *ρ* = 0.01.

Tables 18–25.

**Table 18:** Ebola virus indication.

*ℓ*	Normal	Lung, *ρ* = 0.01	P-value	P-value random
9	100.0	99.99±(< 10^−5^)	1.0	0.5
10	99.94	99.96±0.0005	< 0.01	0.9989
11	98.11	98.61±0.0035	< 0.01	1.0
12	86.99	88.54±0.0080	< 0.01	0.9074
13	56.32	58.74±0.0125	< 0.01	< 0.01
14	23.94	25.48±0.0119	< 0.01	< 0.01
15	7.82	8.41±0.0079	< 0.01	< 0.01
16	2.40	2.57±0.0045	< 0.01	< 0.01
17	0.62	0.68±0.0025	< 0.01	< 0.01
18	0.12	0.14±0.0013	< 0.01	0.0210
*ℓ*	Normal	Bladder, *ρ* = 0.01	P-value	P-value random
9	100.0	99.99±(< 10^−5^)	1.0	0.5
10	99.94	99.96±0.0005	< 0.01	0.8464
11	98.11	98.62±0.0036	< 0.01	1.0
12	86.99	88.54±0.0083	< 0.01	0.8328
13	56.32	58.63±0.0124	< 0.01	< 0.01
14	23.94	25.40±0.0112	< 0.01	< 0.01
15	7.82	8.38±0.0076	< 0.01	< 0.01
16	2.40	2.56±0.0044	< 0.01	< 0.01
17	0.62	0.68±0.0024	< 0.01	< 0.01
18	0.12	0.14±0.0013	< 0.01	< 0.01
*ℓ*	Normal	Mela, *ρ* = 0.01	P-value	P-value random
9	100.0	99.99±(< 10^−5^)	1.0	1.0
10	99.94	99.96±0.0005	< 0.01	0.9999
11	98.11	98.55±0.0035	< 0.01	1.0
12	86.99	88.45±0.0081	< 0.01	1.0
13	56.32	58.78±0.0130	< 0.01	< 0.01
14	23.94	25.58±0.0113	< 0.01	< 0.01
15	7.82	8.46±0.0075	< 0.01	< 0.01
16	2.40	2.59±0.0044	< 0.01	< 0.01
17	0.62	0.68±0.0024	< 0.01	< 0.01
18	0.12	0.14±0.0013	< 0.01	< 0.01

**Table 19:** CMV indication.

*ℓ*	Normal	Lung, *ρ* = 0.01	P-value	P-value random
9	100.0	99.99±(< 10^−5^)	1.0	0.4631
10	99.71	99.84±0.0004	< 0.01	1.0
11	94.88	95.97±0.0017	< 0.01	1.0
12	74.92	77.22±0.0031	< 0.01	1.0
13	43.52	45.49±0.0035	< 0.01	1.0
14	18.64	19.54±0.0029	< 0.01	1.0
15	6.60	6.89±0.0019	< 0.01	1.0
16	2.23	2.31±0.0011	< 0.01	1.0
17	0.77	0.79±0.0007	< 0.01	1.0
18	0.27	0.28±0.0004	< 0.01	1.0
*ℓ*	Normal	Bladder, *ρ* = 0.01	P-value	P-value random
9	100.0	99.99±(< 10^−5^)	1.0	0.1164
10	99.71	99.85±0.0004	< 0.01	0.2682
11	94.88	96.06±0.0017	< 0.01	1.0
12	74.92	77.45±0.0031	< 0.01	1.0
13	43.52	45.67±0.0036	< 0.01	1.0
14	18.64	19.63±0.0029	< 0.01	1.0
15	6.60	6.93±0.0020	< 0.01	1.0
16	2.23	2.33±0.0012	< 0.01	1.0
17	0.77	0.79±0.0007	< 0.01	0.9999
18	0.27	0.28±0.0004	< 0.01	0.9996
*ℓ*	Normal	Mela, *ρ* = 0.01	P-value	P-value random
9	100.0	99.99±(< 10^−5^)	1.0	0.4026
10	99.71	99.83±0.0004	< 0.01	1.0
11	94.88	95.88±0.0017	< 0.01	1.0
12	74.92	76.94±0.0032	< 0.01	1.0
13	43.52	45.20±0.0036	< 0.01	1.0
14	18.64	19.39±0.0029	< 0.01	1.0
15	6.60	6.84±0.0020	< 0.01	1.0
16	2.23	2.30±0.0011	< 0.01	1.0
17	0.77	0.78±0.0007	< 0.01	1.0
18	0.27	0.28±0.0004	< 0.01	0.9999

**Table 20:** Dengue virus indication.

*ℓ*	Normal	Lung, *ρ* = 0.01	P-value	P-value random
9	100.0	99.99±(< 10^−5^)	1.0	0.5
10	100.0	99.98±0.0002	1.0	0.9511
11	99.30	99.45±0.0033	< 0.01	1.0
12	91.65	92.69±0.0094	< 0.01	0.9996
13	64.19	66.20±0.0159	< 0.01	< 0.01
14	29.87	31.21±0.0169	< 0.01	< 0.01
15	9.98	10.52±0.0119	< 0.01	< 0.01
16	2.81	2.99±0.0072	< 0.01	< 0.01
17	0.73	0.78±0.0035	< 0.01	0.0167
18	0.19	0.20±0.0019	< 0.01	0.9490
*ℓ*	Normal	Bladder, *ρ* = 0.01	P-value	P-value random
9	100.0	99.99±(< 10^−5^)	1.0	0.5
10	100.0	99.98±0.0002	1.0	0.3175
11	99.30	99.47±0.0033	< 0.01	0.9999
12	91.65	92.69±0.0094	< 0.01	0.9999
13	64.19	66.12±0.0163	< 0.01	< 0.01
14	29.87	31.12±0.0166	< 0.01	< 0.01
15	9.98	10.49±0.0114	< 0.01	< 0.01
16	2.81	2.97±0.0066	< 0.01	< 0.01
17	0.73	0.78±0.0037	< 0.01	0.6529
18	0.19	0.20±0.0018	< 0.01	0.9154
*ℓ*	Normal	Mela, *ρ* = 0.01	P-value	P-value random
9	100.0	99.99±(< 10^−5^)	1.0	< 0.01
10	100.0	99.98±0.0002	1.0	0.9623
11	99.30	99.43±0.0032	< 0.01	1.0
12	91.65	92.60±0.0094	< 0.01	1.0
13	64.19	66.19±0.0168	< 0.01	< 0.01
14	29.87	31.23±0.0163	< 0.01	< 0.01
15	9.98	10.53±0.0114	< 0.01	< 0.01
16	2.81	2.98±0.0068	< 0.01	< 0.01
17	0.73	0.78±0.0035	< 0.01	0.8850
18	0.19	0.20±0.0019	< 0.01	0.9918

**Table 21:** EBV indication.

*ℓ*	Normal	Lung, *ρ* = 0.01	P-value	P-value random
9	100.0	99.99±(< 10^−5^)	1.0	0.4341
10	99.94	99.97±0.0002	< 0.01	0.9999
11	98.26	98.69±0.0013	< 0.01	1.0
12	86.74	88.26±0.0032	< 0.01	1.0
13	58.29	60.10±0.0047	< 0.01	1.0
14	27.43	28.38±0.0043	< 0.01	1.0
15	10.05	10.34±0.0033	< 0.01	1.0
16	3.19	3.28±0.0025	< 0.01	1.0
17	1.02	1.04±0.0014	< 0.01	1.0
18	0.33	0.33±0.0008	< 0.01	1.0
*ℓ*	Normal	Bladder, *ρ* = 0.01	P-value	P-value random
9	100.0	99.99±(< 10^−5^)	1.0	0.1901
10	99.94	99.97±0.0002	< 0.01	0.2963
11	98.26	98.71±0.0012	< 0.01	1.0
12	86.74	88.35±0.0033	< 0.01	1.0
13	58.29	60.16±0.0046	< 0.01	1.0
14	27.43	28.42±0.0044	< 0.01	1.0
15	10.05	10.37±0.0034	< 0.01	1.0
16	3.19	3.29±0.0024	< 0.01	1.0
17	1.02	1.05±0.0015	< 0.01	1.0
18	0.33	0.33±0.0010	< 0.01	1.0
*ℓ*	Normal	Mela, *ρ* = 0.01	P-value	P-value random
9	100.0	99.99±(< 10^−5^)	1.0	0.3681
10	99.94	99.97±0.0002	< 0.01	1.0
11	98.26	98.66±0.0013	< 0.01	1.0
12	86.74	88.11±0.0033	< 0.01	1.0
13	58.29	59.85±0.0046	< 0.01	1.0
14	27.43	28.16±0.0042	< 0.01	1.0
15	10.05	10.23±0.0033	< 0.01	1.0
16	3.19	3.24±0.0022	< 0.01	1.0
17	1.02	1.03±0.0013	< 0.01	1.0
18	0.33	0.32±0.0008	1.0	1.0

**Table 22:** HHV indication.

*ℓ*	Normal	Lung, *ρ* = 0.01	P-value	P-value random
9	100.0	99.99±(< 10^−5^)	1.0	0.7781
10	99.83	99.90±0.0004	< 0.01	1.0
11	95.96	96.87±0.0020	< 0.01	1.0
12	79.24	81.36±0.0050	< 0.01	1.0
13	49.20	51.48±0.0060	< 0.01	< 0.01
14	21.84	23.18±0.0050	< 0.01	< 0.01
15	7.91	8.42±0.0031	< 0.01	< 0.01
16	2.73	2.89±0.0021	< 0.01	< 0.01
17	1.05	1.09±0.0015	< 0.01	< 0.01
18	0.48	0.49±0.0017	< 0.01	< 0.01
*ℓ*	Normal	Bladder, *ρ* = 0.01	P-value	P-value random
9	100.0	99.99±(< 10^−5^)	1.0	0.5638
10	99.83	99.90±0.0004	< 0.01	0.9999
11	95.96	96.93±0.0021	< 0.01	1.0
12	79.24	81.48±0.0051	< 0.01	1.0
13	49.20	51.50±0.0067	< 0.01	< 0.01
14	21.84	23.15±0.0051	< 0.01	< 0.01
15	7.91	8.41±0.0033	< 0.01	< 0.01
16	2.73	2.89±0.0021	< 0.01	< 0.01
17	1.05	1.10±0.0016	< 0.01	< 0.01
18	0.48	0.49±0.0017	< 0.01	< 0.01
*ℓ*	Normal	Mela, *ρ* = 0.01	P-value	P-value random
9	100.0	99.99±(< 10^−5^)	1.0	0.8672
10	99.83	99.89±0.0004	< 0.01	1.0
11	95.96	96.79±0.0021	< 0.01	1.0
12	79.24	81.22±0.0052	< 0.01	1.0
13	49.20	51.50±0.0069	< 0.01	< 0.01
14	21.84	23.26±0.0055	< 0.01	< 0.01
15	7.91	8.48±0.0034	< 0.01	< 0.01
16	2.73	2.91±0.0022	< 0.01	< 0.01
17	1.05	1.11±0.0015	< 0.01	< 0.01
18	0.48	0.49±0.0017	< 0.01	< 0.01

**Table 23:** HPV indication.

*ℓ*	Normal	Lung, *ρ* = 0.01	P-value	P-value random
9	100.0	99.98±(< 10^−5^)	1.0	0.1587
10	99.97	99.96±0.0007	1.0	0.9984
11	99.05	99.23±0.0039	< 0.01	0.9999
12	90.48	91.75±0.0114	< 0.01	< 0.01
13	61.24	63.56±0.0183	< 0.01	< 0.01
14	28.22	29.77±0.0175	< 0.01	< 0.01
15	9.84	10.55±0.0124	< 0.01	< 0.01
16	3.19	3.45±0.0076	< 0.01	< 0.01
17	1.10	1.19±0.0046	< 0.01	< 0.01
18	0.50	0.53±0.0025	< 0.01	< 0.01
*ℓ*	Normal	Bladder, *ρ* = 0.01	P-value	P-value random
9	100.0	99.98±(< 10^−5^)	1.0	0.5
10	99.97	99.97±0.0007	1.0	0.9724
11	99.05	99.24±0.0039	< 0.01	0.9999
12	90.48	91.71±0.0111	< 0.01	< 0.01
13	61.24	63.34±0.0182	< 0.01	< 0.01
14	28.22	29.65±0.0180	< 0.01	< 0.01
15	9.84	10.48±0.0121	< 0.01	< 0.01
16	3.19	3.43±0.0072	< 0.01	< 0.01
17	1.10	1.17±0.0042	< 0.01	< 0.01
18	0.50	0.52±0.0024	< 0.01	0.5377
*ℓ*	Normal	Mela, *ρ* = 0.01	P-value	P-value random
9	100.0	99.98±(< 10^−5^)	1.0	0.1587
10	99.97	99.96±0.0007	1.0	0.9999
11	99.05	99.21±0.0038	< 0.01	1.0
12	90.48	91.77±0.0115	< 0.01	< 0.01
13	61.24	63.70±0.0181	< 0.01	< 0.01
14	28.22	29.96±0.0179	< 0.01	< 0.01
15	9.84	10.64±0.0124	< 0.01	< 0.01
16	3.19	3.48±0.0072	< 0.01	< 0.01
17	1.10	1.19±0.0043	< 0.01	< 0.01
18	0.50	0.53±0.0025	< 0.01	< 0.01

**Table 24:** Measles virus indication.

*ℓ*	Normal	Lung, *ρ* = 0.01	P-value	P-value random
9	100.0	99.99±(< 10^−5^)	1.0	0.5
10	99.97	99.98±0.0004	< 0.01	0.9998
11	98.23	98.70±0.0037	< 0.01	1.0
12	86.15	87.87±0.0094	< 0.01	0.9934
13	54.27	56.70±0.0140	< 0.01	< 0.01
14	22.64	23.98±0.0127	< 0.01	< 0.01
15	7.29	7.73±0.0084	< 0.01	< 0.01
16	2.19	2.29±0.0049	< 0.01	0.5766
17	0.68	0.69±0.0027	< 0.01	0.3373
18	0.19	0.19±0.0013	0.5424	0.0179
*ℓ*	Normal	Bladder, *ρ* = 0.01	P-value	P-value random
9	100.0	99.99±(< 10^−5^)	1.0	0.5
10	99.97	99.98±0.0004	< 0.01	0.9992
11	98.23	98.71±0.0037	< 0.01	1.0
12	86.15	87.88±0.0091	< 0.01	0.3703
13	54.27	56.57±0.0138	< 0.01	< 0.01
14	22.64	23.86±0.0124	< 0.01	< 0.01
15	7.29	7.69±0.0078	< 0.01	0.5972
16	2.19	2.28±0.0049	< 0.01	0.9990
17	0.68	0.69±0.0026	< 0.01	0.7406
18	0.19	0.19±0.0014	0.5426	0.0220
*ℓ*	Normal	Mela, *ρ* = 0.01	P-value	P-value random
9	100.0	99.99±(< 10^−5^)	1.0	1.0
10	99.97	99.98±0.0004	< 0.01	0.9999
11	98.23	98.65±0.0038	< 0.01	1.0
12	86.15	87.81±0.0096	< 0.01	1.0
13	54.27	56.61±0.0131	< 0.01	< 0.01
14	22.64	23.92±0.0121	< 0.01	< 0.01
15	7.29	7.71±0.0082	< 0.01	0.0134
16	2.19	2.27±0.0048	< 0.01	0.9999
17	0.68	0.68±0.0026	0.0137	0.9609
18	0.19	0.19±0.0014	0.9999	0.9017

**Table 25:** Yellow fever virus indication.

*ℓ*	Normal	Lung, *ρ* = 0.01	P-value	P-value random
9	100.0	99.99±(< 10^−5^)	1.0	0.5
10	100.0	99.98±0.0002	1.0	0.6664
11	99.05	99.30±0.0034	< 0.01	1.0
12	92.10	93.10±0.0094	< 0.01	1.0
13	65.28	67.19±0.0169	< 0.01	< 0.01
14	30.54	31.81±0.0176	< 0.01	< 0.01
15	10.81	11.26±0.0119	< 0.01	< 0.01
16	3.15	3.32±0.0070	< 0.01	0.3854
17	0.87	0.94±0.0039	< 0.01	< 0.01
18	0.29	0.31±0.0022	< 0.01	< 0.01
*ℓ*	Normal	Bladder, *ρ* = 0.01	P-value	P-value random
9	100.0	99.99±(< 10^−5^)	1.0	0.5
10	100.0	99.98±0.0003	1.0	0.9609
11	99.05	99.30±0.0034	< 0.01	0.9999
12	92.10	93.11±0.0096	< 0.01	0.9999
13	65.28	67.09±0.0173	< 0.01	0.9999
14	30.54	31.75±0.0164	< 0.01	0.0112
15	10.81	11.25±0.0116	< 0.01	0.0579
16	3.15	3.32±0.0068	< 0.01	0.1432
17	0.87	0.94±0.0038	< 0.01	< 0.01
18	0.29	0.32±0.0023	< 0.01	< 0.01
*ℓ*	Normal	Mela, *ρ* = 0.01	P-value	P-value random
9	100.0	99.99±(< 10^−5^)	1.0	< 0.01
10	100.0	99.98±0.0003	1.0	0.9888
11	99.05	99.26±0.0036	< 0.01	1.0
12	92.10	93.01±0.0090	< 0.01	1.0
13	65.28	67.03±0.0170	< 0.01	1.0
14	30.54	31.77±0.0166	< 0.01	< 0.01
15	10.81	11.26±0.0121	< 0.01	< 0.01
16	3.15	3.33±0.0073	< 0.01	< 0.01
17	0.87	0.95±0.0040	< 0.01	< 0.01
18	0.29	0.32±0.0023	< 0.01	< 0.01

### A.6 Error probability data for Fig 6

To compute $E|\{i:{\tilde{a}}_{i}\neq a_{i}\}|$ in (2) we proceeded as follows. We have

$$\begin{array}{c} E|\left\{i:{\tilde{a}}_{i}\neq a_{i}\right\}|=\sum_{i}P\left({\tilde{a}}_{i}\neq a_{i}\right) \end{array}$$ (7)

where the summation ranges over amino acid positions. Let us compute $P({\tilde{a}}_{1}\neq a_{1})$—for the other terms we proceed in the same way. Observe that *a*_1_ is a function of the first three nucleotides *y*_1_, *y*_2_, *y*_3_ of the normal exome *y*. To emphasize this, let us write *a*_1_ as *a*_1_(*y*_1_, *y*_2_, *y*_3_). Similarly, ${\tilde{a}}_{1}$ is a function of the first three nucleotides ${\tilde{y}}_{1},{\tilde{y}}_{2},{\tilde{y}}_{3}$ of the cancer genome $\tilde{y}$ and we write it as ${\tilde{a}}_{1}\left({\tilde{y}}_{1},{\tilde{y}}_{2},{\tilde{y}}_{3}\right)$. Therefore, we have

$$\begin{array}{c} P\left({\tilde{a}}_{1}\neq a_{1}\right)=\underset{{\tilde{a}}_{1}\left({\tilde{y}}_{1},{\tilde{y}}_{2},{\tilde{y}}_{3}\right)\neq a_{1}\left(y_{1},y_{2},y_{3}\right)}{\sum_{({\tilde{y}}_{1},{\tilde{y}}_{2},{\tilde{y}}_{3}):}}\prod_{j=1}^{3}P_{c,\rho }\left({\tilde{y}}_{j}|y_{j}\right) \end{array}$$ (8)

where $P_{c,\rho }\left({\tilde{y}}_{j}|y_{j}\right)$ is the cancer channel defined in the Appendix A.2.
